# 90K/*LGALS3BP* expression is upregulated in COVID-19 but may not restrict SARS-CoV-2 infection

**DOI:** 10.1007/s10238-023-01077-2

**Published:** 2023-05-10

**Authors:** Laure Bosquillon de Jarcy, Bengisu Akbil, Baxolele Mhlekude, Johanna Leyens, Dylan Postmus, Greta Harnisch, Jenny Jansen, Marie L. Schmidt, Annette Aigner, Fabian Pott, Robert Lorenz Chua, Lilian Krist, Roberta Gentile, Barbara Mühlemann, Terence C. Jones, Daniela Niemeyer, Julia Fricke, Thomas Keil, Tobias Pischon, Jürgen Janke, Christian Conrad, Stefano Iacobelli, Christian Drosten, Victor M. Corman, Markus Ralser, Roland Eils, Florian Kurth, Leif Sander, Christine Goffinet

**Affiliations:** 1 Institute of Virology, Campus Charité Mitte, Charité - Universitätsmedizin Berlin, Charitéplatz 1, 10117 Berlin, Germany; 2 Berlin Institute of Health at Charité – Universitätsmedizin Berlin, Charitéplatz 1, 10117 Berlin, Germany; 3 Speciality Network: Infectious Diseases and Respiratory Medicine, Charité - Universitätsmedizin Berlin, Charitéplatz 1, 10117 Berlin, Germany; 4 Institute of Biometry and Clinical Epidemiology, Charité - Universitätsmedizin Berlin, Charitéplatz 1, 10117 Berlin, Germany; 5 Center for Digital Health, Berlin Institute of Health (BIH) at Charité – Universitätsmedizin Berlin, Berlin, Germany, Charitéplatz 1, 10117 Berlin, Germany; 6 Institute of Social Medicine, Epidemiology and Health Economics, Charité - Universitätsmedizin Berlin, Charitéplatz 1, 10117 Berlin, Germany; 7 MediaPharma SrL, 66013 Chieti, Italy; 8 Department of Zoology, Centre for Pathogen Evolution, University of Cambridge, Downing St., Cambridge, CB2 3EJ UK; 9 German Center for Infection Research, Associated Partner Charité, Berlin, Germany; 10 Institute of Clinical Epidemiology and Biometry, University of Würzburg, Josef-Schneiderstr. 2, 97080 Würzburg, Germany; 11 State Institute of Health, Bavarian Health and Food Safety Authority, Eggenreuther Weg 43, 91058 Erlangen, Germany; 12 Molecular Epidemiology Research Group, Max-Delbrueck-Center for Molecular Medicine in the Helmholtz Association (MDC), 13125 Berlin, Germany; 13 Biobank Technology Platform, Max-Delbrueck-Center for Molecular Medicine in the Helmholtz Association (MDC), 13125 Berlin, Germany; 14 Core Facility Biobank, Berlin Institute of Health at Charité - Universitätsmedizin Berlin, 10178 Berlin, Germany; 15 Charité - Universitätsmedizin Berlin, Corporate Member of Freie Universität Berlin and Humboldt-Universität Zu Berlin, 10117 Berlin, Germany; 16 Department of Biochemistry, Charité - Universitätsmedizin Berlin, Charitéplatz 1, 10117 Berlin, Germany; 17 Molecular Biology of Metabolism Laboratory, The Francis Crick Institute, London, NW11AT UK; 18 German Center for Lung Research (DZL), 35392 Gießen, Germany; 19 Health Data Science Unit, Heidelberg University Hospital and BioQuant, 69120 Heidelberg, Germany; 20 Department of Tropical Medicine, Bernhard Nocht Institute for Tropical Medicine, 20359 Hamburg, Germany; 21 Department of Medicine, University Medical Center, Hamburg-Eppendorf, 20251 Hamburg, Germany

**Keywords:** SARS-CoV-2, COVID-19, 90K, LGALS3BP, Interferon

## Abstract

Glycoprotein 90K, encoded by the interferon-stimulated gene *LGALS3BP*, displays broad antiviral activity. It reduces HIV-1 infectivity by interfering with Env maturation and virion incorporation, and increases survival of Influenza A virus-infected mice via antiviral innate immune signaling. Its antiviral potential in SARS-CoV-2 infection remains largely unknown. Here, we analyzed the expression of 90K/*LGALS3BP* in 44 hospitalized COVID-19 patients at multiple levels. We quantified 90K protein concentrations in serum and PBMCs as well as *LGALS3BP* mRNA levels. Complementary, we analyzed two single cell RNA-sequencing datasets for expression of *LGALS3BP* in respiratory specimens and PBMCs from COVID-19 patients. Finally, we analyzed the potential of 90K to interfere with SARS-CoV-2 infection of HEK293T/ACE2, Calu-3 and Caco-2 cells using authentic virus. 90K protein serum concentrations were significantly elevated in COVID-19 patients compared to uninfected sex- and age-matched controls. Furthermore, PBMC-associated concentrations of 90K protein were overall reduced by SARS-CoV-2 infection in vivo, suggesting enhanced secretion into the extracellular space. Mining of published PBMC scRNA-seq datasets uncovered monocyte-specific induction of *LGALS3BP* mRNA expression in COVID-19 patients. In functional assays, neither 90K overexpression in susceptible cell lines nor exogenous addition of purified 90K consistently inhibited SARS-CoV-2 infection. Our data suggests that 90K/*LGALS3BP* contributes to the global type I IFN response during SARS-CoV-2 infection in vivo without displaying detectable antiviral properties in vitro.

**Supplementary Information:**

The online version contains supplementary material available at 10.1007/s10238-023-01077-2.

## Background

SARS-CoV-2 infects host cells via interaction of its spike protein with the ACE2 receptor [1, 2]. The infectivity of SARS-CoV-2 particles is largely determined by the characteristics of their spike protein. Antiviral strategies targeting its biosynthesis, maturation, and fusion activity may be clinically beneficial. Membrane fusion-mediating viral proteins are targeted by antiviral proteins expressed from interferon (IFN)-stimulated genes (ISGs). 90K (gene name *LGALS3BP*) is a ubiquitously expressed cellular secreted glycoprotein with multiple antiviral activities. Expression of *LGALS3BP* is stimulated by IFNs, resulting in upregulated 90K serum concentrations in individuals with viral infections, including HIV-1 [3–5], HCV [6, 7], hantaviruses [8], and dengue virus [9]. In the context of HIV-1 infection, 90K expressed in virus-producing cells inhibits proper proteolytic processing of the envelope protein precursor and virion incorporation of glycoproteins, resulting in reduction of particle infectivity [10]. 90K has also been suggested to inhibit virion production through inhibition of HIV-1 Gag trafficking [11]. In addition, 90K activates signaling to mount a cell-intrinsic antiviral profile that was essential for survival of experimental influenza virus infection in mice [12]. Furthermore, secreted 90K may promote NK cell activity, CD8^+^ T-cell-mediated cytotoxicity, and cytokine production [13]. Defective IFN signaling, due to inborn mutations in type I IFN-mediated immunity [14] and presence of autoantibodies against type I IFN [15, 16] have been reported as risk factors for critical COVID-19. Given the IFN-stimulated manner of *LGALS3BP* expression and its reported association with viral infections in vivo, including its potential suitability as a prognostic marker for disease progression in HIV-1/AIDS [17], we investigated the expression profile of 90K/*LGALS3BP* in specimens of COVID-19 patients and uninfected individuals and probed for a potential direct antiviral role of 90K against SARS-CoV-2 infection.

## Methods

### COVID-19 cohort

Hospitalized COVID-19 patients’ blood samples and clinical data were collected at Charité - Universitätsmedizin Berlin in the context of the *Pa-COVID-19 Study* [18]. *Pa-COVID-19* is a prospective observational cohort study collecting data longitudinally from patients with confirmed COVID-19 weekly during their hospitalization. Data include, inter alia, epidemiological and demographic parameters, medical history, clinical course and morbidity [18]. Patients analyzed in this study were sampled between March and November 2020. All patients provided a positive SARS-CoV-2 RT-PCR from respiratory specimens. The study was approved by the ethics committee of Charité (EA2/066/20). Written informed consent was obtained from all patients or legal representatives. In this work, we analyzed 44 COVID-19 patients. 42 provided serum samples and 13 blood samples for peripheral blood mononuclear cell (PBMC) isolation. Most patients were sampled longitudinally (Suppl. Table S1). To minimize confounding issues, patients with conditions known to enhance 90K serum concentrations were excluded from our study: asthma bronchiale [19], history of malignoma within the last ten years [20], Hepatitis B, C, or liver cirrhosis [21], or HIV-1 [10]. Furthermore, we excluded patients with acute herpes zoster as modulation of ISGs is reported in this context [22].

### Classification of disease severity

We assessed COVID-19 disease severity using the World Health Organization (WHO) ordinal scale for clinical improvement on sampling day [23].

- WHO 1: Asymptomatic infection.
- WHO 2: Symptomatic infection, ambulatory care.
- WHO 3: Hospitalization, no supplemental oxygen required.
- WHO 4: Supplemental oxygen required.
- WHO 5: High Flow or Continuous Positive Airway Pressure (CPAP) required.
- WHO 6: Mechanical ventilation required.
- WHO 7: Extracorporeal Membrane Oxygenation (ECMO) and/or Continuous Renal
  Replacement Therapy (CRRT) required.
- WHO 8: Death.

For practical reasons, we designated patients admitted to general hospital wards as “mild COVID-19” (WHO 3-4) and patients admitted to intensive care units as “severe COVID-19” (WHO 5-7). Within the latter, patients receiving mechanical ventilation, ECMO or CRRT were designated as “critical COVID-19” (WHO 6-7). Patients with multiple WHO grades within the sampling period (6 out of 42 patients) were assigned the highest disease severity grade.

### Healthy controls

A control population was provided by the study center Berlin-Mitte of the German National Cohort (Gesundheitsstudie, NAKO*—*Germany’s largest population-based longitudinal cohort study [24–26]. The study aims to determine novel and better characterize known risk and protection factors for respiratory and infectious diseases, cardiovascular diseases, cancer, diabetes, neurodegenerative and psychiatric diseases as well as musculoskeletal diseases in a random sample of the general population. Between 2014 and 2019, a total of 205,415 men and women aged 19–74 years were recruited and examined in 18 study centers in Germany. The baseline assessment included a face-to-face interview, self-administered questionnaires and a wide range of biomedical examinations. Biomaterials were collected from all participants including serum, EDTA plasma, buffy coats, RNA and erythrocytes, urine, saliva, nasal swabs and stool [27]. 42 serum samples from uninfected individuals were selected randomly from participants recruited in the study region (Berlin), after exclusion of potentially confounding conditions that may lead to an elevation of 90K serum levels, as specified in the study patients with COVID-19. To rule out SARS-CoV-2 infection in the controls, we chose serum samples collected between 2014 and 2015. Samples were requested according to Use & Access regulations of the NAKO. Control samples were manually matched 1:1 to our COVID-19 patients for sex and age categories: < 25 years, 25–34 years, 35–44 years, 45–54 years, 55–64 years, ≥ 65 years. Details on assignment of healthy controls to COVID-19 patients are provided in Suppl. Table S2. Retrieval of referred blood samples was approved by the ethics committee of Charité—Universitätsmedizin Berlin (EA1/076/13 vom 28.3.2014).

Retrieval of further blood samples and cell isolation from healthy anonymized donors was conducted with approval of the local ethics committee (Ethical review committee of Charité Berlin, vote EA4/120/22).

### Cells

Calu-3 (ATCC HTB-55), Caco-2 (ATCC HTB-37), Vero E6 (ATCC CRL-1586), and HEK293T (ATCC CRL-3216) cells were cultured in DMEM (Sigma Aldrich) with 4.5 g/L glucose, supplemented with 10% fetal bovine serum, 1% penicillin/streptomycin, and 1% L-glutamine at 37 °C and 5% CO_2_. HEK293T/ACE2 cells were additionally supplemented with 13 µg/ml Blasticidin. Caco-2/90K cells were additionally supplemented with 10 µg/ml Puromycin.

PBMCs from COVID-19 patients and healthy controls from anonymized blood donors were isolated from 2–3 ml EDTA whole blood. Samples were mixed 1:1 with PBS and centrifuged on Pancoll (Pan Biotech) for 30 min at 200 × *g*. PBMCs were then washed with PBS. Remaining erythrocytes were lysed with ACK-Lysis Buffer (8,29 g NH_4_Cl, 1 g KHCO_3_, 0,0367 g EDTA, 600 ml H_2_O), followed by PBS washing. PBMC pellets were frozen at − 20 °C.

### Virus, lenti- and retroviral particles

SARS-CoV-2 strain Munich 984 (strain: SARS-CoV-2/human/DEU/BavPat2-ChVir984, NCBI GenBank Acc. No. MT270112.1) was propagated on Vero E6 cells and concentrated using Vivaspin^®^ 20 concentrators (Sartorius Stedim Biotech). Virus stocks were diluted in OptiPro serum-free medium supplemented with 0.5% gelatine and PBS and stored at − 80 °C. Infectious titer was defined by plaque titration assay.

VSV-G-pseudotyped lentiviral vector particles encoding human 90K were generated by calcium phosphate-based transfection of HEK293T cells with the packaging plasmid pCMV ΔR8.91 [28], the lentiviral transfer plasmid pWPI-puro-90K-myc or pWPI puro (gift of Thomas Pietschmann) and pCMV-VSV-G [29]. The plasmid pWPI-puro-90K-myc was generated by standard molecular cloning. VSV-G-pseudotyped retroviral vector particles encoding human ACE2 were generated by calcium phosphate-based transfection of HEK293T cells with the packaging plasmid MLV gag-pol [30], the MLV-based transfer plasmid pCX4bsrACE2 [31] and pCMV-VSV-G. Vector-containing supernatant was collected 40 and 64 h post-transfection and subjected to ultracentrifugation through a 20% sucrose cushion. Aliquots were stored at − 80 °C.

### Infection with authentic SARS-CoV-2

Calu-3, Caco-2, and HEK293T/ACE2 cells were seeded in 6-well plates at densities of 6 × 10^5^, 3 × 10^5^, and 4.5 × 10^5^ cells/ml, respectively. Cells were infected with SARS-CoV-2 (MOI 0.01). Virus inoculum was removed after one hour, cells were washed with PBS and resupplied with fresh medium. Where indicated, cells were pretreated with Remdesivir (20 µM), and treatment was continued until the end of the experiment.

### Data presentation and statistical analysis

Bar graphs indicate mean values and error bars indicate standard deviation. Whiskers in box plots indicate minimum to maximum values. Trends over time are displayed using locally weighted scatterplot smoothing (LOESS) estimates.

To determine statistical significance between two independent groups, we used unpaired t-tests and paired t-tests when comparing cases and matched controls, assuming normal distribution. To analyze data with both independent and dependent observations due to longitudinal measurements of an individual, we used mixed effects models.

Based on scRNA-seq data we used these models to compare expression between cases and controls, just as between mild cases, severe cases, and controls regarding ten cell types from PBMCs and 20 cell types from respiratory samples. As we consider all analyses to be exploratory, we did not adjust for multiple testing.

When analyzing independent observations, graphs and statistical analyses were generated using *Graph Pad Prism 9.1.2.* Further statistical analyses were performed using R. The code utilized for the analysis will be available at https://github.com/GoffinetLab/SARS-CoV-2_90K_patient_study.

## Results

### Serum 90K concentrations are elevated in COVID-19 patients as compared to healthy controls

**Fig. 1:**
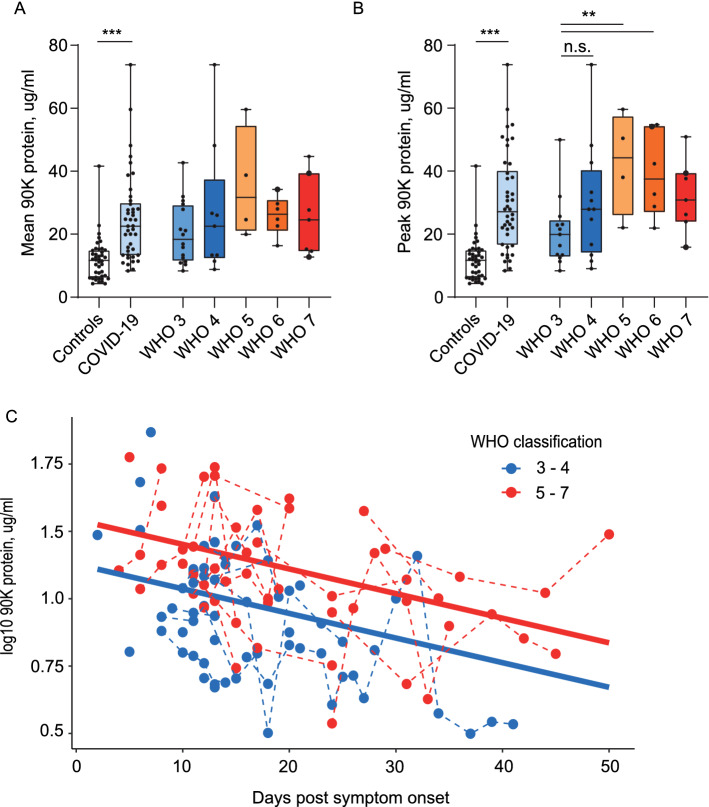
Serum 90K Concentrations are elevated in COVID-19 Patients. **A** Mean 90K serum concentrations in COVID-19 compared to controls. Controls n = 42, COVID-19: n = 42, WHO 3: n = 13, WHO 4: n = 12, WHO 5: n = 4, WHO 6: n = 6, WHO 7: n = 7. Multiple ELISA quantifications per patient are presented with the mean, large points indicate deceased patients. Cases versus controls *p* < 0.0001 and differences between WHO groups (reference WHO 3) were assessed using linear mixed effects models. **B** Peak 90K serum concentrations in COVID-19. Cohort and dot legend identical to Fig. 1A. Paired t-test cases versus matched controls (*p* < 0.0001), unpaired t-test WHO 3 versus 4 *p* = 0.14, 3 versus 5 *p* = 0.007, 3 versus 6 *p* = 0.006, 3 versus 7 *p* = 0.045. **C** 90K serum concentrations over time in COVID-19 patients, log_10_. n = 41/116 (individuals/time points), WHO 3-4: n = 24/62, WHO 5-7: n = 17/54. 1 of 42 patients is not depicted due to asymptomatic disease course. Log-normalized concentrations were modeled using a linear mixed effects model (marginal R^2^ = 0.21; conditional R^2^ = 0.56). The effects of “days post symptom onset” and “WHO classification” were statistically significant (*p* = 0.0004 and *p* = 0.008 respectively). Regression lines are shown, with solid lines indicating the predictions on the population level and dashed lines connecting subjects

We quantified 90K concentrations in 119 serum samples from 42 hospitalized COVID-19 patients and 42 age- and sex-matched healthy controls (NAKO). Both mean (Fig. 1A) and peak (Fig. 1B) 90K serum concentrations were significantly higher in patients with SARS-CoV-2 compared to controls (all *p* < 0.0001). Highest 90K concentrations were detected in patients classified WHO 5 (WHO 3 vs. 5, *p* = 0.052 for mean 90K, *p* = 0.007 for peak 90K). Analyzing all samples with respect to the sampling time point, we saw gradually decreasing 90K levels over time with highest levels at early infection stages (linear mixed effects model, *p* < 0.001) and similar rate of decline in patients with mild and severe disease (Fig. 1C). Overall, patients with severe COVID-19 (WHO 5-7) had higher 90K serum concentrations than patients with mild COVID-19 (WHO 3-4) when considering symptom onset (linear mixed effects model *p* = 0.008).

To identify a potential influence of dexamethasone treatment on 90K serum levels, we compared patients treated before and after introduction of dexamethasone as standard of care for COVID-19 patients with oxygen supply [32]. No significant differences between both groups were detectable (Suppl. Fig. S1).

Furthermore, we analyzed possible interrelations between 90K serum concentrations, viral RNA concentrations from nasopharyngeal swabs and anti-SARS-CoV-2 IgG/IgA titers and found no association (Suppl. Fig. S2A, B). For demographic aspects, no sex-dependent differences in 90K serum levels were noted (Suppl. Fig. S2C), but we recorded higher concentrations in older healthy individuals > 55 y compared to younger individuals (linear mixed effects model *p* = 0.027, Suppl. Fig. S2D). This difference was not observed in COVID-19 patients, which displayed enhanced 90K serum concentrations irrespective of age.

### PBMC-associated 90K protein concentrations are reduced in the context of SARS-CoV-2 infection

**Fig. 2:**
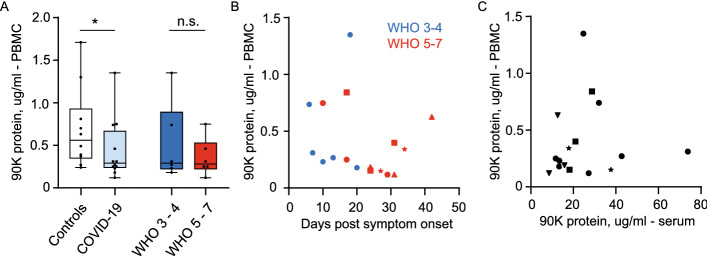
PBMC-associated 90K Protein Concentrations are reduced in COVID-19. **A** 90K protein in PBMCs, one mean value per individual. Controls: n = 10, COVID-19: n = 12/17 (individuals/timepoints), WHO 3-4: n = 6/6, WHO 5-7: n = 6/11. Linear mixed effects model log_10_ norm (Controls vs. COVID-19) *p* = 0.047 and (WHO 3-4 vs. WHO 5-7) *p* = 0.39. **B** 90K protein in PBMCs over time in COVID-19. Cohort identical to 2A. Dots show individuals with a single sampling time point. Other symbols show values belonging to longitudinally sampled individuals. **C** 90K protein in PBMCs and sera at identical timepoints in COVID-19 (both samples taken with maximum interval of 48 h) n = 11/16 (individuals/time points). Dot legend s.2B

We hypothesized that 90K serum protein originates from peripheral blood cells. Since antiviral functions have been described for intra- and extracellular 90K protein, we aimed at determining if SARS-CoV-2 infection in vivo increases 90K synthesis, or whether 90K secretion or stability may be modulated. Therefore, we quantified cell-associated 90K protein from lysed PBMCs (17 samples from 12 COVID-19 patients) and found reduced concentrations in COVID-19 compared to healthy controls, regardless of disease severity (linear mixed effects model, *p* = 0.047, Fig. 2A). This suggests an enhanced secretion or extracellular stability of 90K in COVID-19 rather than an overall enhanced biosynthesis in PBCMs. Furthermore, in contrast to serum 90K, we obtained no evidence for time-dependent changes of cell-associated 90K (Fig. 2B) or association with respective serum concentrations in single individuals (Fig. 2C).

### *LGALS3BP* mRNA expression in PBMCs is unaltered in COVID-19

**Fig. 3:**
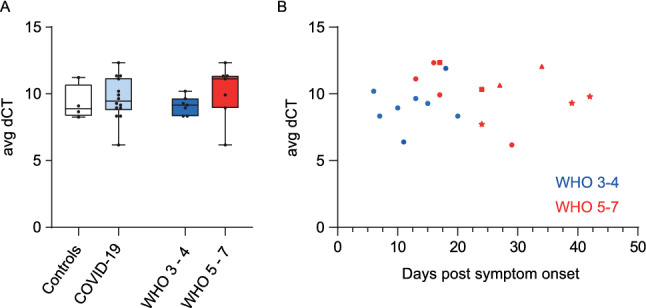
LGALS3BP mRNA Expression in PBMCs is Unaltered in COVID-19. **A** *LGALS3BP* mRNA Expression in PBMCs. Controls: n = 4, COVID-19 cohort: n = 14, WHO 3-4: n = 7 and WHO 5-7: n = 7, one mean value per individual. **B** *LGALS3BP* mRNA expression in PBMCs over time. Controls: n = 4, COVID-19 cohort: n = 14/19 (individuals/time points), WHO 3-4: n = 7/8, WHO 5-7: n = 7/11

PBMCs of our COVID-19 cohort (19 samples from n = 14 individuals) showed no upregulated *LGALS3BP* mRNA expression compared to healthy controls (Fig. 3A) and no time-dependent expression changes (Fig. 3B), as judged by qRT-PCR analysis in bulk PBMCs.

In summary, in our cohort of SARS-CoV-2-infected individuals, 90K protein concentrations were enhanced in sera and reduced in PBMCs, while *LGALS3BP* mRNA expression in PBMCs from COVID-19 patients remained unaltered as compared to healthy controls.

### Single cell RNA-sequencing data hint towards upregulated *LGALS3BP* mRNA expression in monocytes, dendritic cells and plasmablasts of COVID-19 patients

To identify potential cell type-specific *LGALS3BP* mRNA expression patterns in individual cell types of PBMCs of COVID-19 patients that may be undetectable in our whole-PBMC analysis (Fig. 3), we analyzed published scRNA-seq datasets from a Dual Center Cohort Study [33]. As the scRNA-seq data of both cohorts had been generated by different experimental approaches, we analyzed each cohort separately.

**Fig. 4:**
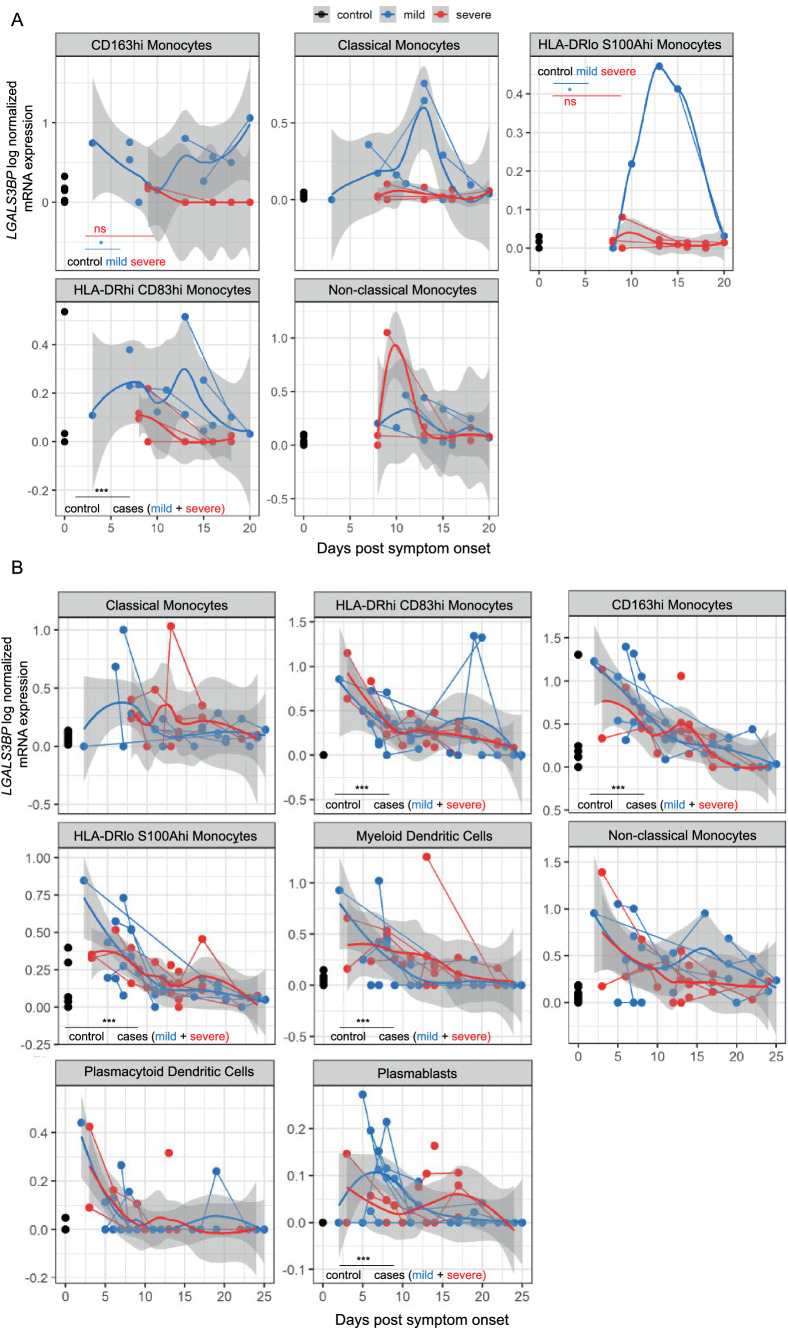
Single cell RNA-sequencing data hint towards upregulated *LGALS3BP* mRNA Expression in Monocytes, Dendritic Cells and Plasmablasts of COVID-19 Patients. **A**: Cohort A, **B**: Cohort B. *LGALS3BP* log normalized mRNA expression over time. Uninfected controls (black), “mild” COVID-19 (blue) and “severe” COVID-19 (red). Thick lines indicate smoothed population trends based on LOESS estimate, thin lines connect subjects. Shaded areas indicate 95% confidence interval (CI) for the LOESS estimate. Data are derived from a preceding dual center cohort study [33]. Further information regarding the subdivision in cohort A and B is provided in the supplemental methods section. **A** CD163^hi^ monocytes control: n = 19/19, mild: n = 7/10, severe: n = 4/6 (individuals/time points)*,* classical monocytes control: n = 22/22, mild: n = 6/13, severe: n = 10/14, HLA-DR^Io^ S100A^hi^ monocytes control: n = 8/8, mild: n = 4/5, severe: n = 10/14**,** HLA-DR^hi^ CD83^hi^ monocytes control: n = 9/9, mild: n = 6/13, severe: n = 10/12, non-classical monocytes control: n = 22/22, mild: n = 6/10, severe: n = 6/13. **B** Classical monocytes—control: n = 13/13, mild: n = 7/17, severe: n = 8/20, HLA-DR^hi^ CD83^hi^ monocytes—control: n = 12/12, mild: n = 8/22, severe: n = 9/28, CD163^hi^ monocytes—control: n = 13/13, mild: n = 8/21, severe: n = 9/26, HLA-DR^Io^ S100A^hi^ monocytes control: n = 13/13, mild: n = 8/22, severe: n = 9/28, mDCs control: n = 13, mild: n = 8/22, severe: n = 9/23, non-classical monocytes—control: n = 13/13, mild: n = 8/22, severe: n = 9/23, plasmacytoid dendritic cells (pDCs) control: n = 12/12, mild: n = 8/21, severe: n = 9/24, plasmablasts control: n = 12, mild: n = 8/22, severe: n = 9/28

In both COVID-19 cohorts, we detected an overall increase of *LGALS3BP* expression in several monocyte fractions compared to healthy controls (Fig. 4, Suppl. Fig. S7). In cohort B, *LGALS3BP* expression levels were, in addition, significantly elevated in dendritic cells and plasmablasts (mixed linear regression models, *p* < 0.001). Overall, expression was highest at early infection stages and decreased after symptom onset (*p* < 0.01, Fig. 4, Suppl. Fig. S7). Interestingly, we identified low to absent *LGALS3BP* expression levels in CD163^hi^ and HLA-DR^lo^ S100A^hi^ monocytes of cohort A in severe COVID-19 (Fig. 4A) while patients with mild COVID-19 showed elevated levels compared to controls. However, this finding was not reproduced in cohort B.

### Expression of *LGALS3BP* mRNA is not upregulated in respiratory samples from COVID-19 patients

We analyzed the expression of *LGALS3BP* in scRNA-seq data from 32 respiratory samples originating from a previous scRNA-seq study [34]. In contrast to the expression profile in PBMCs, *LGALS3BP* expression was not increased, neither in epithelial nor in immune cell types of respiratory COVID-19 samples (Suppl. Fig. S4).

### SARS-CoV-2 infection fails to induce *LGALS3BP* expression in a lung-derived cell line

**Fig. 5:**
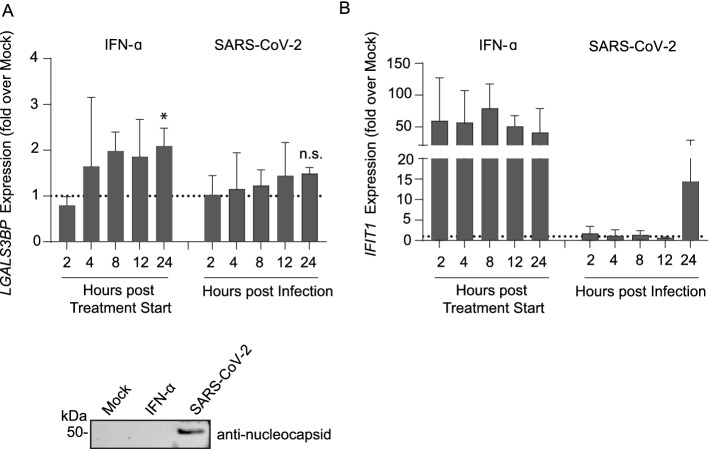
SARS-CoV-2 Infection Fails to Induce *LGALS3BP* Expression in a Lung-derived Cell Line. Calu-3 cells were treated with 500 IU/ml IFN-α2a or infected with SARS-CoV-2 (MOI 0.01) and harvested two-24 h later for *LGALS3BP* (**A**) and *IFIT1* (**B**) mRNA qRT-PCR. Values are normalized to the first mock condition (harvested at two hours) of each experiment. 24 h post infection or IFN-α2a treatment, equal volumes of supernatant were harvested and loaded for immunoblotting. Results are derived from three independent experiments. Statistical significance was determined using an unpaired t-test (mock vs. IFN-α2a *p* = 0.038, mock vs. SARS-CoV-2 *p* = 0.29)

To elucidate SARS-CoV-2’s ability to induce *LGALS3BP* expression, we infected Calu-3 cells with authentic SARS-CoV-2. We reached infection rates of 19% 48 h post infection as measured by flow cytometry (Suppl. Fig. S8A). *LGALS3BP* expression was slightly induced after IFN-α2 treatment (twofold induction compared to mock, *p* = 0.038) but not after SARS-CoV-2 infection (Fig. 5A). To demonstrate the overall effectiveness of IFN-α2a treatment, we assessed the expression of a prototypic ISG, *IFIT-1,* which displayed distinctly upregulated expression levels after IFN-α2a treatment (Fig. 5B). Taken together, upregulation of neither *LGALS3BP* nor *IFIT1* mRNA was detectable in Calu-3 cells after infection with SARS-CoV-2, in line with SARS-CoV-2’s ability to prevent efficient recognition by cell-intrinsic innate immunity [35]. We consider that the moderate increase of *IFIT-1* mRNA at 24 h post infection in SARS-CoV-2 infected Calu-3 cells results from an outlying data point rather than representing a genuine upregulation of *IFIT-1* mRNA.

### Heterologous 90K expression mildly inhibits SARS-CoV-2 infection of ACE2-expressing HEK293T cells but not Caco-2 cells

**Fig. 6:**
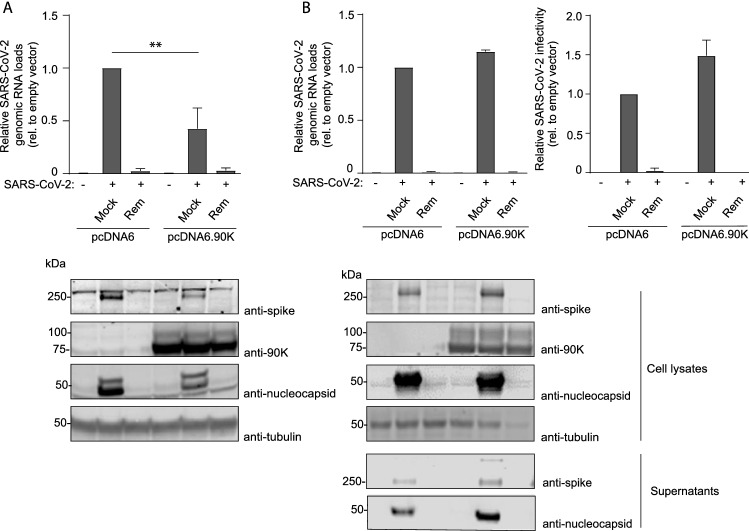
Heterologous 90K Expression Mildly Inhibits SARS-CoV-2 Infection of ACE2-expressing HEK293T cells but Not Caco-2 Cells. **A** SARS-CoV-2 genome equivalents in supernatants of HEK293T/ACE2 cells. Cells were mock-infected or infected in the absence or presence of Remdesivir. 90K-myc was expressed via transient transfection. 24 h post SARS-CoV-2 infection (MOI 0.01), supernatants were harvested for quantification of SARS-CoV-2 genome equivalents. Statistical significance was determined using an unpaired t-test (*p* = 0.007). Immunoblot of cell lysates was performed using indicated antibodies. **B** SARS-CoV-2 genome equivalents from qRT-PCR and infectivity from plaque assays from supernatants of infected Caco-2 cells. 90K-myc expression in Caco-2 cells was achieved by lentiviral transduction. 24 h post SARS-CoV-2 infection (MOI 0.01), supernatant was harvested for quantification of SARS-CoV-2 genome equivalents and released infectivity. Immunoblot of supernatants and cell lysates was performed using indicated antibodies. Results are derived from two to three independent experiments

To identify a potential antiviral activity of 90K, we conducted experiments in HEK293T cells that are devoid of detectable 90K protein expression [10, 36] and therefore suitable for heterologous overexpression of 90K. SARS-CoV-2 RNA concentrations in the culture supernatant of infected ACE2-expressing HEK293T cells were mildly (2.5-fold) reduced in the context of expression of 90K-myc. Absence of detectable PFUs and viral proteins in the supernatants of infected HEK293T/ACE2 cells (independent of the 90K expression status, data not shown) precluded analysis of the role of 90K on the infectivity and composition of released virions. Intracellular spike and nucleocapsid expression was diminished 1.7-fold and 3.3-fold, respectively (Fig. 6A). Interestingly, 90K expression seems to be accompanied by reduced relative levels of processed spike protein within virus-producing cells, in analogy to 90K-imposed interference with the cleavage of the HIV-1 glycoprotein precursor [10]. Efficient 90K expression was confirmed by immunoblotting.

We next aimed at verifying these findings in a SARS-CoV-2-susceptible cell line that expresses endogenous ACE2 and in which endogenous 90K protein is undetectable by immunoblotting. Therefore, we stably transduced Caco-2 cells with empty vector or 90K-myc and infected them with SARS-CoV-2. We reached infection rates of 23–32% 24 h post infection as measured by flow cytometry (Suppl. Fig. S8B). In contrast to our findings in HEK293T/ACE2 cells, overexpression of 90K in Caco-2 cells neither reduced the concentration of viral RNA, nor diminished the expression of nucleocapsid and spike in SARS-CoV-2 producing cells, nor influenced spike processing (Fig. 6B). Importantly, the infectivity of released particles that originated from 90K-expressing Caco-2 cells was intact. Pre-treatment with the viral polymerase inhibitor Remdesivir efficiently reduced infection of both cell lines (Fig. 6A and B), as expected.

To analyze a potential role of exogenously added 90K protein on SARS-CoV-2 infection, we pretreated Calu-3 and Caco-2 cells with 90K, for which signaling via cell surface proteins has been reported [13, 37–39] or the D2 domain of 90K, given the ability of the latter to promote cell adhesion [40]. Pretreatment of cells, or treatment of virus stocks with purified 90K protein or D2 domain of 90K, failed to modulate release and infectivity of SARS-CoV-2 particles (Suppl. Fig. S6).

## Discussion

Our findings of upregulated 90K serum concentrations in COVID-19 with highest levels in severe disease courses are in line with proteomic and serological findings from hospitalized COVID-19 patients [41, 42]. Overall reduced PBMC-associated 90K protein concentrations in SARS-CoV-2-infected individuals and, conversely, upregulated *LGALS3BP* mRNA in monocytes, plasmablasts, and dendritic cells hint towards a complex, compartment- and cell type-specific regulation of 90K expression in SARS-CoV-2 infection in vivo. However, data for PBMC-associated 90K protein levels were gathered from a rather small cohort and should ideally be confirmed in a larger sample size study. It is conceivable that elevated serum 90K concentrations in COVID-19 originate from monocytes, even though our data do not exclude other 90K-producing cell types as alternative sources. Strikingly, scRNA-seq data from respiratory samples of COVID-19 patients showed no upregulated *LGALS3BP* mRNA expression, illustrating the multifaceted ability of SARS-CoV-2 to antagonize or evade cellular sensing machineries in productively infected cells [43, 44].

The role of type I IFN in the context of COVID-19 has been discussed controversially and may be time point-dependent. On the one hand, overdriven IFN responses are thought to cause severe COVID-19 [45, 46]. Since *LGALS3BP* is an ISG, enhanced 90K serum levels in patients with severe COVID-19 could possibly reflect exaggerated type I IFN responses. On the other hand, mounting of an efficient IFN-mediated antiviral state is associated with effective clearance of SARS-CoV-2 infection and milder course of the disease [47]. According to the latter, patients with critical COVID-19 (WHO 6, 7) within the severe disease group (WHO 5-7) had a trend towards lower 90K serum levels than patients classified WHO 5. Similarly, some monocyte fractions of severely ill COVID-19 patients failed to show enhanced *LGALS3BP* upregulation in our scRNA-seq analysis (cohort A), whereas mildly affected patients displayed distinct upregulation. However, this pattern could not be reproduced in cohort B, possibly due to technical differences in the scRNA-seq pipelines and/or unknown clinical differences such as medication or comorbidities. Nevertheless, since *LGALS3BP* is a prototypic ISG [10, 48], its upregulation might be merely indicative of the overall mounting of an effective antiviral gene expression program to which *LGALS3BP* is part of, and does not necessarily imply that 90K is an active antiviral component of SARS-CoV-2 infection.

In line with this idea, we did not generate consistent evidence for an anti-SARS-CoV-2 activity of 90K that is exerted directly on SARS-CoV-2 infection. Mild inhibitory effects were detected in HEK293T/ACE2 cells heterologously expressing 90K, complementing former results suggesting a reduced susceptibility to transduction with lentiviral particles decorated with SARS-CoV-2 spike in the same cell line overexpressing 90K [49]. The inability of HEK293T/ACE2 cells to release infectious SARS-CoV-2 precluded to analyze if the mild reduction of viral RNA secretion and diminished spike processing is accompanied by a potentially more pronounced infectivity defect that has been reported in the context of HIV-1 production [10]. However, no evidence for 90K-imposed restriction was detected in infected Caco-2 cells. Of note, the latter cell line expresses endogenous ACE2 and is fully permissive to SARS-CoV-2 infection, and thus represents a superior cell line model over HEK293T/ACE2 cells. Finally, absent modulation of infection upon exogenous addition of soluble 90K protein argues against a major contribution of 90K signaling via cell surface receptors [13, 37–40] to control of SARS-CoV-2 infection in Calu-3 cells. It is conceivable that 90K’s potentially existing antiviral properties are antagonized by a virus-encoded component, a possibility that should be analyzed in future studies. Taken together, our cell culture experiments with infectious SARS-CoV-2 failed to identify a consistent inhibitory role of 90K in the context of wild-type virus expressing all non-structural protein-coding genes.

In summary, our study describes the expression pattern of *LGALS3BP* in COVID-19 at multiple levels. We propose that 90K/*LGALS3BP* contributes to the global type I IFN response during SARS-CoV-2 infection in vivo without exerting biologically relevant, direct antiviral effects detectable in cell culture. Therefore, further investigations on 90K as a potential biomarker and/or potential immunomodulator of SARS-CoV-2 pathogenesis are warranted.

### Supplementary Information

Below is the link to the electronic supplementary material.

Supplementary file1 (DOCX 1883 KB)
