# Supplementary material for: 90K/LGALS3BP expression is upregulated in COVID-19 but may not restrict SARS-CoV-2 infection
Source: Clin Exp Med. 2023 May 10;23(7):3689–700. doi: 10.1007/s10238-023-01077-2 (PMC10170455; doi:10.1007/s10238-023-01077-2)
Supplement: Supplementary file 1 — Supplementary file1 (DOCX 1883 KB) [file 10238_2023_1077_MOESM1_ESM.docx]

**Supplement**

**Supplemental Methods**

**Analysis of Single Cell RNA-Sequencing Data of PBMCs**

Analyzed single cell RNA-sequencing (scRNA-seq) data from PBMCs originate from a published Dual Center Cohort Study [[33]](https://paperpile.com/c/oA8C2P/Tldj). Two independent patient cohorts had previously been recruited between March and July 2020 at two university medical centers in Germany. Cohort A comprises PBMC samples from Charité - Universitätsmedizin Berlin collected within the *Pa-COVID-19* study [[18]](https://paperpile.com/c/oA8C2P/a1Ej) (27 samples from 18 SARS-CoV-2 patients - mild (WHO 2-4): n=8, severe (WHO 5-7): n=10, and 22 healthy controls). Data had been generated by scRNA-seq with a droplet-based platform. CellRanger v3.1.0 (10x Genomics) was used to process scRNA-seq. To generate a digital gene expression (DGE) matrix for each sample, we mapped their reads to a combined reference of GRCh38 genome and SARS-CoV-2 genome and recorded the number of UMIs for each gene in each cell [[33]](https://paperpile.com/c/oA8C2P/Tldj). Cohort B comprises PBMC samples collected at University Hospital Bonn (50 samples from 17 SARS-CoV-2 patients - mild: n=8, severe: n=9, 13 healthy controls) and scRNA-seq data had been generated by a microwell-based system. STAR 2.6.1b was used for alignment against the Gencode v27 reference genome [[50]](https://paperpile.com/c/oA8C2P/tymG). Dropseq-tools 2.0.0 were used to quantify gene expression and collapse to UMI count data (<https://github.com/broadinstitute/Drop-seq/>). For hashtag-oligo based demultiplexing of single-cell transcriptomes and subsequent assignment of cell barcodes to their sample of origin the respective multiplexing tag sequences were added to the reference genome and quantified as well [[33]](https://paperpile.com/c/oA8C2P/Tldj). As the scRNA-seq data of both cohorts had been generated by different experimental approaches, we analyzed each cohort separately in this study. Pre-processed scRNA-seq datasets were obtained from the European Genome Archive (EGA) under access number EGAS00001004571 and data were extracted using the FetchData function of the R package Seurat [[51]](https://paperpile.com/c/oA8C2P/iHbv).

**Analysis of Single Cell RNA-Sequencing Data of Respiratory Samples**

We analyzed a published dataset of 32 respiratory samples from 19 hospitalized COVID-19 patients and five healthy controls for *LGALS3BP* expression, originating from a published scRNA-seq study [[34]](https://paperpile.com/c/oA8C2P/YVxL). The study was designed to investigate the immune response in patients with COVID-19 by scRNA-seq of nasopharyngeal and bronchial samples to identify molecular correlates of disease severity. All patients were enrolled either in the Pa-COVID-19 study at Charité - Universitätsmedizin Berlin [[18]](https://paperpile.com/c/oA8C2P/a1Ej) or the prospective SC2-Study at University Hospital Leipzig [[34]](https://paperpile.com/c/oA8C2P/YVxL), which both included all patients with COVID-19 who were hospitalized between March 11 and May 7, 2020, at either hospital. Analyzed samples had been obtained through nasopharyngeal swabs, bronchial protected specimen brushes, and bronchoalveolar lavages and had been processed with a droplet-based system. Amongst the 19 COVID-19 patients, eight had a moderate disease course (WHO 3), while eleven had a critical disease course (WHO 6-7). The transcripts were aligned to a customized reference genome in which the SARS-CoV-2 genome (Refseq-ID: NC_045512) was added as an additional chromosome to the human reference genome hg19 (10X Genomics, version 3.0.0). An entry summarizing the entire SARS-CoV-2 genome as one ‘gene’ was appended to the hg19 annotation gtf file, and the genome was indexed using ‘cellranger_mkref ’ [[33]](https://paperpile.com/c/oA8C2P/Tldj). The pre-processed scRNA-seq dataset was obtained from the European Genome Archive (EGA) under access number EGAS00001004481 and data were extracted using the *FetchData* function of the R package Seurat [[51]](https://paperpile.com/c/oA8C2P/iHbv)*.*

**Transfection**

HEK293T/ACE2 cells were transfected with empty vector (pcDNA.6myc) or pcDNA.90K-myc [[10]](https://paperpile.com/c/oA8C2P/1Cfwn) by calcium-phosphate transfection using TaKaRa CalPhos™ Mammalian Transfection Kit following the manufacturer's instructions.

**Reagents**

Roferon-A (IFN-α2a) was purchased from Roche (Basel, Switzerland) and used at a concentration of 500 IU/ml. Remdesivir (Gilead Sciences) was kindly provided by the Department of Infectious Diseases and Respiratory Medicine, Charité - Universitätsmedizin Berlin and used at a concentration of 10 µM.

**Purification of 90K and 90K-D2**

Individual cDNAs encoding full 90K and e 90K-D2 were generated by polymerase chain reaction (PCR). D2 is defined as a 28 kDa fragment corresponding to amino acid residues 134-288 of 90K. Fragments were subcloned into an Evitria’s proprietary vector system (EVITRIA, Schlieren, Switzerland) allowing fusion in-frame to the 6xHis tag sequence C-terminally to the domain.

90K and 90K-D2 were transiently expressed by transfection into Chinese Hamster Ovary (CHO) cells. Concentrated culture supernatants were applied to a column of Ni-NTA Superflow (Qiagen) and proteins were eluted with increasing concentrations of imidazole according to the manufacturer’s instructions.

**SARS-CoV-2 Plaque Titration Assay**

The infectious titer was calculated via plaque titration assay. Vero E6 cells were plated at 3.5 x 10^5^ cell/ml in 24 wells and infected with 200 µl of a serial dilution of virus-containing cell culture supernatant diluted in OptiPro serum-free medium. One hour after adsorption, supernatants were removed and cells overlaid with 2.4% Avicel (FMC BioPolymers) mixed 1:1 in 2 x DMEM. Three days post-infection, the overlay was removed, cells were fixed in 6% formaldehyde and stained with a 0.2% crystal violet, 2% ethanol and 10% formaldehyde.

**Anti-90K ELISA**

*Sera.* Sera were analyzed with the Thermo Fisher Scientific s90K/Mac-2BP ELISA Kit according to the manufacturer’s instructions. Sera were analyzed in duplicates in 1:100 and 1:1000 dilutions.

*PBMCs.* Frozen PBMC pellets (1,5 - 9 x 10^6^ cells) were lysed in 1% Triton X-100 and applied to Thermo Fisher Scientific s90K/Mac-2BP ELISA Kit following the manufacturer’s instructions in a 1:14 dilution. PBMCs were analyzed in single measurements as the amount of patient material was not sufficient for analysis in duplicates.

**Anti-SARS-CoV-2 IgA/IgG ELISA**

Human sera were examined for presence of specific antibodies (IgG and IgA) to the S1 subdomain of The SARS-CoV-2 spike protein using an ELISA kit (Euroimmun, Lübeck, Germany) as described before [[52,53]](https://paperpile.com/c/oA8C2P/kNDMb+7xG8). Samples were tested at a 1:101 dilution and results were considered positive above an optical density (OD) ratio of 1.1. Automated measurement was performed with Euroimmun Analyzer I.

**Viral RNA Load Analysis from Nasopharyngeal Swabs**

SARS-CoV-2 RNA quantification was performed by real-time RT-PCR from upper respiratory tract swabs obtained within standard care. RNA concentrations were quantified by RT-qPCR targeting SARS-CoV-2 E gene [[54]](https://paperpile.com/c/oA8C2P/AbpWG) and are given as logarithm base 10 of the number of RNA copies (viral load) per ml using an empirical formula derived from testing standard curves of SARS-CoV-2 RNA and cell culture supernatants [[55]](https://paperpile.com/c/oA8C2P/y3clc).

**qRT-PCR**

Total RNA extraction from PBMCs and cell lines was performed with Qiagen RNeasy Micro Kit according to the manufacturer’s instructions. RNA isolation was followed by 2-step PCR. Quantification was performed by RT-qPCR in Roche LightCycler 480 II using TaqMan PCR technology with a premade primer-probe kit for *LGALS3BP, MX2*, and *IFIT1* (Applied Biosystems). For *LGALS3BP*, oligonucleotide primers 5′-GCTTCCTTCCTCTCTGCAATGA-3′ (forward), 5′-TCAGGTGAGTAGGGCGACATC-3′ (reverse), 5′-FAM-CTTCAACAACCGGCCAC-TAMRA-3′ (fluorescent probe) were used. For *MX2* and *IFIT1*, we used assay IDs Hs01550814_m1 and Hs01911452_s1, respectively (Thermo Fisher Scientific Waltham, Massachusetts, USA).

Relative mRNA levels were determined using the ΔCT method, with human *RNASEP* mRNA (Applied Biosystems) as internal reference. Each sample was analyzed in triplicate. Viral RNA extraction was performed using Macherey Nagel Nucleospin RNA Virus Mini Kit. SARS-CoV-2 genome equivalents were quantified by RT-qPCR targeting SARS-CoV-2 E gene using the following primers: E_Sarbeco_F: ACAGGTACGTTAATAGTTAATAGCGT; E_Sarbeco_P1: FAM-ACACTAGCCATCCTTACTGCGCTTCG-BBQ; E_Sarbeco_R: ATATTGCAGCAGTACGCACACA [[54]](https://paperpile.com/c/oA8C2P/AbpWG). RT-qPCRs were performed using the Superscript III OneStep RT-PCR kit (Invitrogen, Darmstadt, Germany). Absolute quantification was performed using SARS-CoV-2-specific *in vitro*-transcribed RNA standards. Data analysis was performed using Roche LightCycler 480 1.5.1.62SP3 software.

**Immunoblotting**

Cell lysates were generated with M-PER Mammalian Protein Extraction Reagent (Thermo Fisher Scientific, Waltham, Massachusetts, USA). For virus-containing supernatant, 1x SDS-buffer was added to the sample. Proteins were separated on a 10% SDS-PAGE and transferred onto nitrocellulose using a semi-dry transfer system (Bio-Rad Laboratories, Hercules, California, USA). Membranes were blocked with 5% milk powder solution for one hour and incubated overnight with the following primary antibodies: rabbit anti-myc (71D10, Cell Signaling Technology, Danvers, USA), goat anti-Galectin-3BP/MAC-2BP (AF2226, R&D Systems, Minnesota, USA), goat anti-MX2 (sc-47197, Santa Cruz Biotechnology, California, USA), mouse anti-IFIT1 (CF500948, Origene, Maryland, USA), rabbit anti-nucleocapsid (GTX135361, Biozol, Eching, Germany), mouse anti-ACE2 (10108-MM36, Sino Biological, Beijing, China), rabbit anti-SARS Spike (NB100-56578, Novus Biologicals, Minneapolis, USA)**,** rabbit anti-α-tubulin (#2144, Cell Signaling Technology, Massachusetts, USA), and mouse-anti human actin (#8226, Abcam, Cambridge, UK). Secondary antibodies conjugated to Alexa 680/800 fluorescent dyes were used for detection and quantification by Odyssey Infrared Imaging System (LI-COR Biosciences Lincoln, NE, USA).

**Flow Cytometry**

Cells were fixed in 4% PFA (Carl Roth) and permeabilized with 0.1% Triton X-100 (Thermo Fisher Scientific) in PBS before immunostaining with the following antibodies: rabbit anti-nucleocapsid (GTX135361, Biozol, Eching, Germany) as described [[56]](https://paperpile.com/c/oA8C2P/Wy2e), goat anti-Galectin-3BP/MAC-2BP (AF2226, R&D Systems, Minnesota, USA), and mouse anti-ACE2 (10108-MM36, Sino Biological, Beijing, China). Secondary antibodies conjugated to Alexa Fluor 488, 647 (1:1,500; Invitrogen) were used for detection. Flow cytometry analysis was performed using FACS Celesta with BD Diva Software (BD Biosciences) and FlowJo V10 Software (FlowJo).


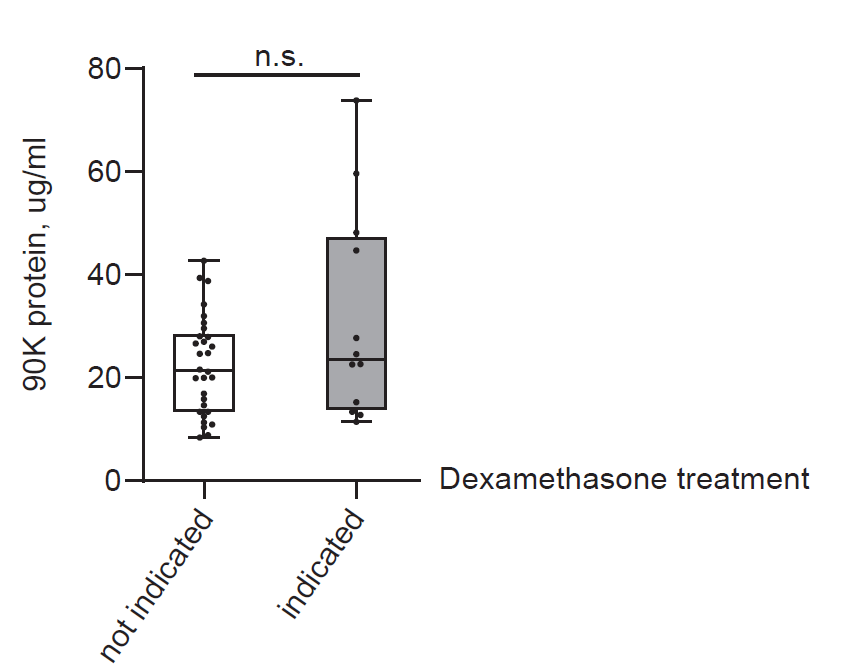


***Suppl. Fig. 1* 90K Serum Concentrations Do Not Depend on Dexamethasone Treatment in our COVID-19 Cohort**

90K protein concentrations in serum according to assumed dexamethasone treatment. Dexamethasone was introduced as standard of care for COVID-19 patients with need for oxygen supply in July 2020 [[32]](https://paperpile.com/c/oA8C2P/PJ28). Patients assigned to the white bar (n=30) were treated for COVID-19 before introduction of dexamethasone as standard of care or, if treated later, presented no need for oxygen supply (WHO 3) and therefore had no indication for dexamethasone treatment. Patients assigned to the gray bar (n=12) were treated from July 2020 on and received oxygen supply (WHO 4 - 7) with indication for dexamethasone treatment. One mean value per patient is depicted. The effect of assumed dexamethasone treatment status on 90K serum levels was assessed using a linear mixed effects model (p=0.07).

**
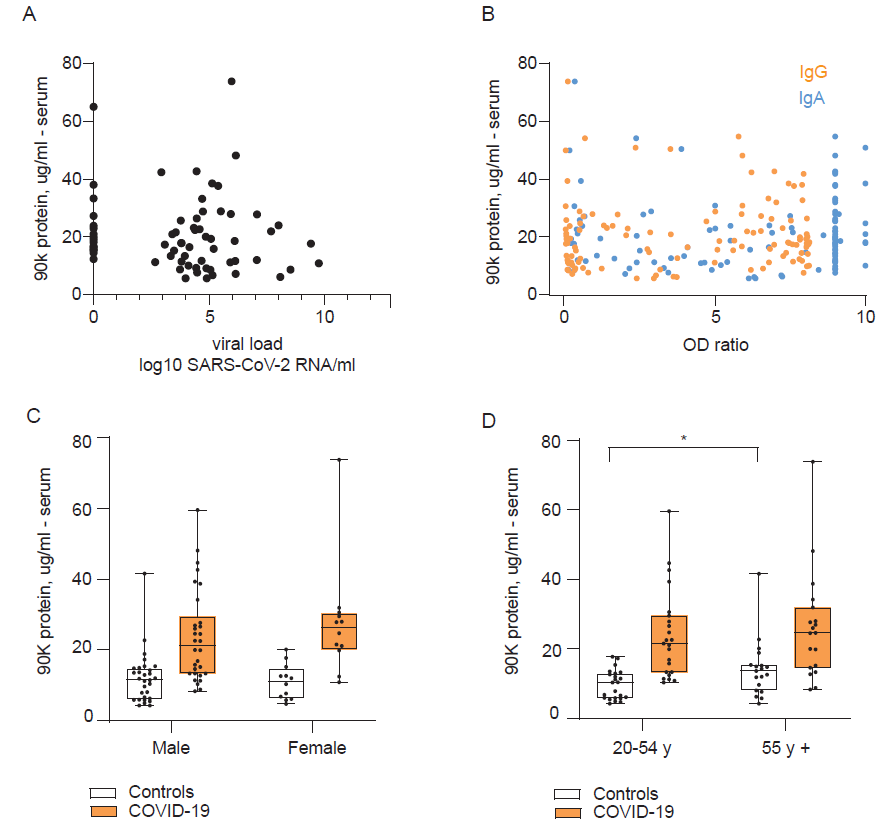
**

***Suppl. Fig. 2* 90K Serum Concentrations in Relation to Infection Status, Antibody Response to SARS-CoV-2, and Demographic Characteristics**

(A) 90K serum protein concentrations and respective SARS-CoV-2 RNA concentrations from nasopharyngeal swabs in COVID-19 patients, n=32 individuals, 68 observations at different time points.

(B) 90K serum protein concentrations and respective IgG/IgA anti-SARS-CoV-2 Spike antibody levels in COVID-19 patients, n=41 individuals, 117 observations at different time points.

(C) 90K protein concentrations in serum and sex of COVID-19 patients and age-matched healthy controls. Male n=30, female n=12, 1 mean value per patient is depicted. The effect of sex on 90K serum concentrations was assessed separately for COVID-19 patients and healthy controls using a linear mixed effects model. This was non-significant in both groups (p=0.39 in COVID-19, p=0.65 in healthy controls).

(D) 90K protein concentrations in serum and age of COVID-19 patients and sex-matched healthy controls. 20-55y n=23, > 55y n=19, 1 mean value per patient is depicted. The effect of age on 90K serum concentrations was assessed separately for COVID-19 patients and healthy controls using a linear mixed effects model. This was non-significant in COVID-19 (p=0.50). For healthy controls this was significant (p=0.027).

**
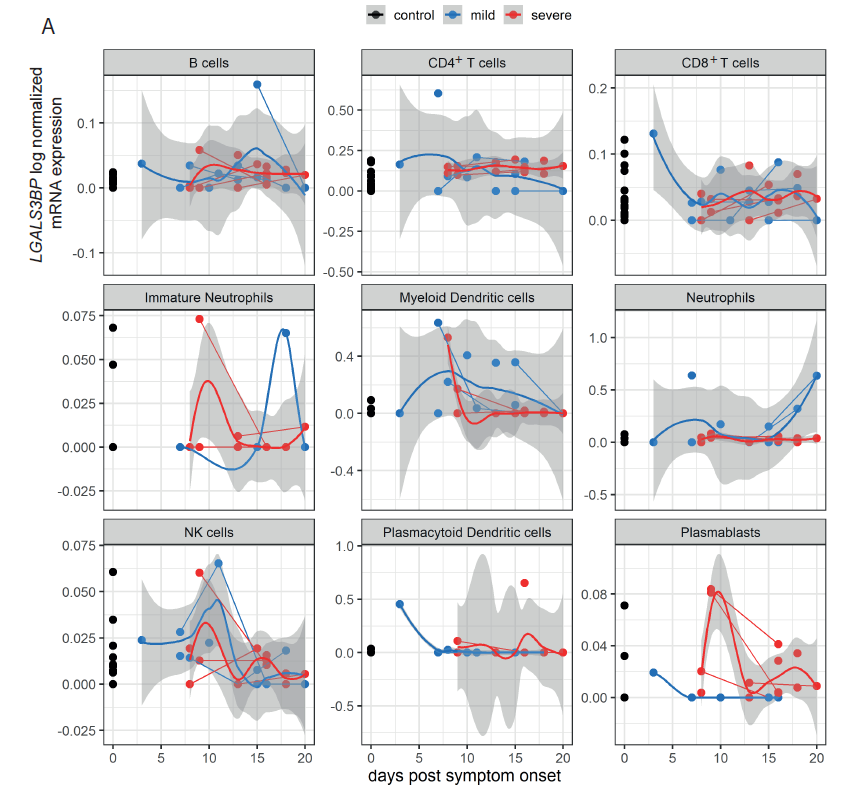
**

**
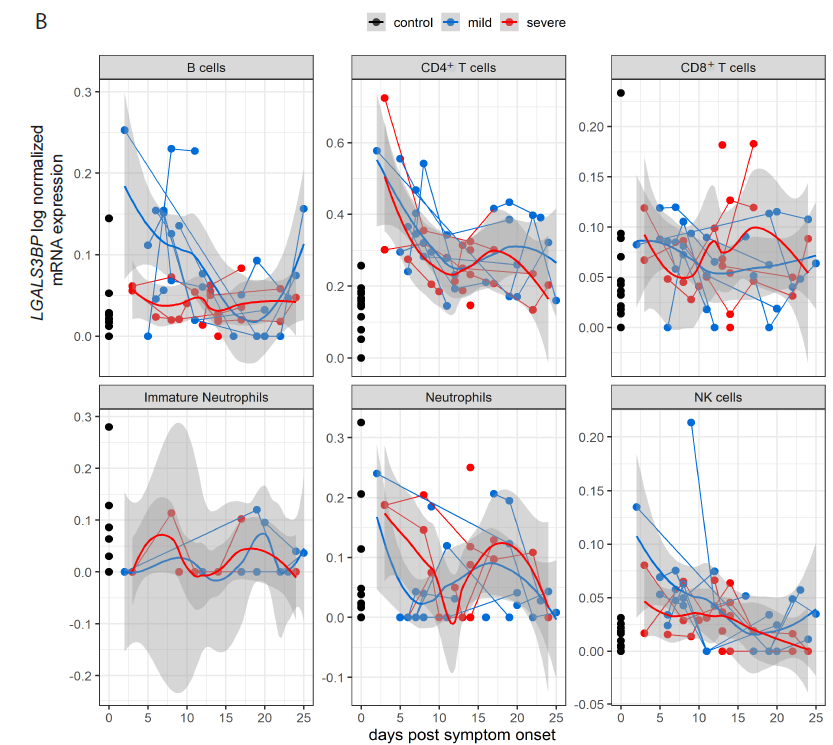
**

***Suppl. Fig. 3 LGALS3BP* scRNA-seq Analysis from PBMCs, Further Cell Types**

**A**: Cohort A, **B**: Cohort B.

*LGALS3BP* log-normalized expression, assigned to days after symptom onset for each sample. Both cohorts are divided into three groups: Uninfected controls (black), “mild” COVID-19 (defined as WHO grade 2-4 in corresponding publication) (blue) and “severe” COVID-19 (WHO grade 5-7 in corresponding publication) (red). Thick lines indicate smoothed population trends based on a LOESS estimate, thin lines connect subjects. shaded areas indicate 95% CI for the LOESS estimate.

(A) B cells control n=22/22, mild n=8/13, severe n=10/14, CD4^+^ T cells control n=22/22, mild n=8/13, severe n=10/14 , CD8^+^ T cells control n=22/22, mild n=8/13, severe n=10/14, immature neutrophils control n=9/9, mild n=4/4, severe n=10/14, myeloid dendritic cells (mDCs) control n=22/22, mild n=8/13, severe n=10/14, neutrophils control n=19/19 (individuals/time points), mild n=8/11, severe n=10/14, NK cells control n=22/22, mild n=8/13, severe n=10/14 , plasmacytoid dendritic cells (pDCs) control n=22/22, mild n=7/12, severe n=9/14, plasmablasts control n=20/20, mild n=5/6, severe n=10/14

(B) B cells control n=13/13, mild n=8/22, severe n=9/28, CD4^+^ T cells control n=13/13, mild n=8/22, severe n=9/28, CD8^+^ T cells control n=13/13, mild n=8/22, severe n=9/28, immature neutrophils control n=12/12, mild n=5/13, severe n=4/10, neutrophils control n=13/13, mild n=8/21, severe n=9/27, NK cells control n=13/13, mild n=8/22, severe n=9/28.


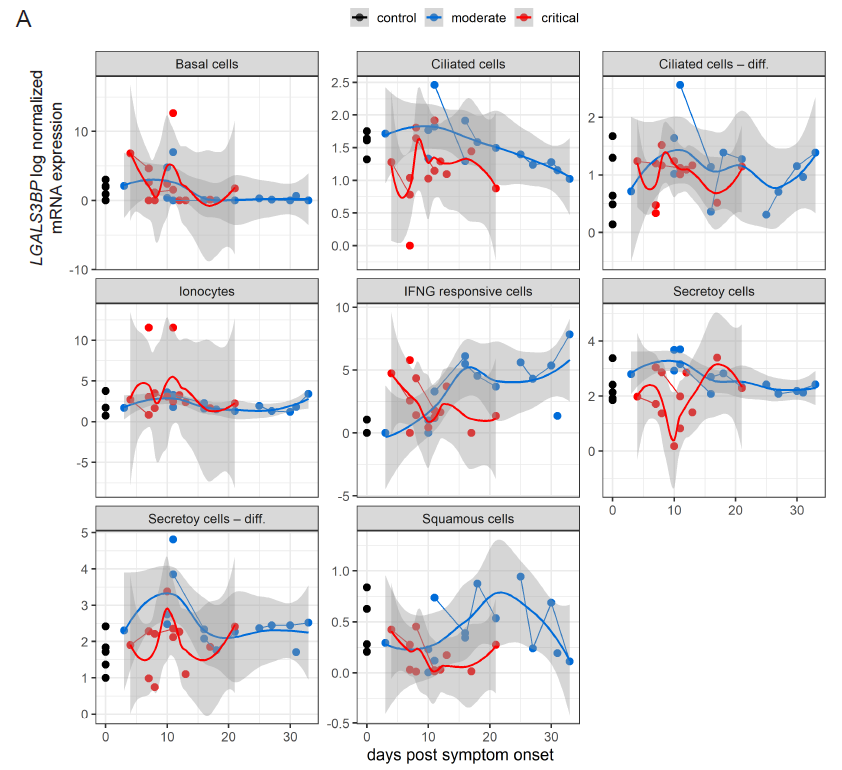


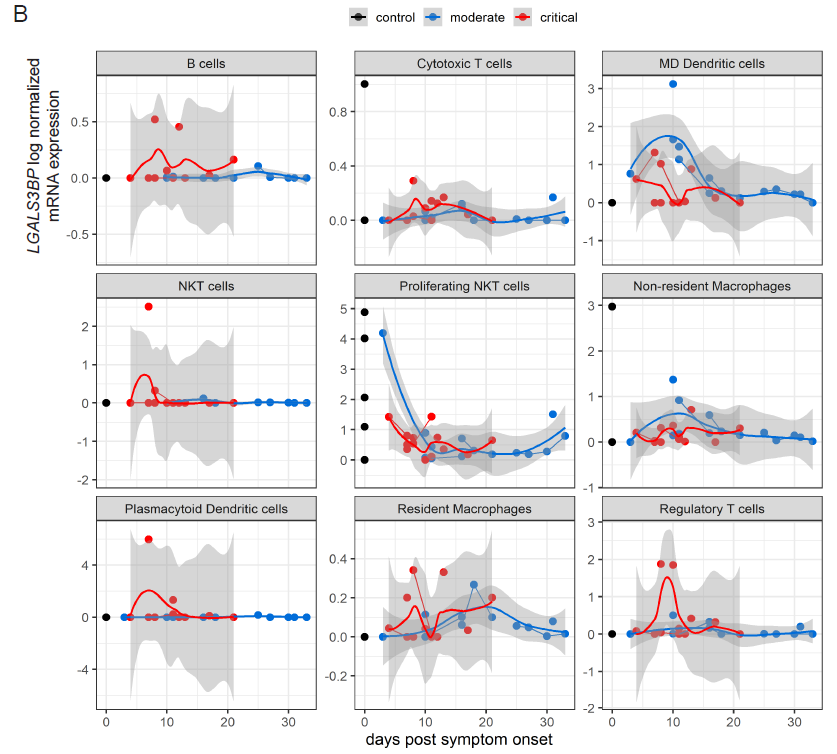


***Suppl. Fig. 4* *LGALS3BP* scRNA-seq Analysis from Respiratory Samples**

**A**: Epithelial cells **B**: Immune cells

*LGALS3B*P log-normalized expression at days after symptom onset for each sample. Both cohorts are divided into three groups: Uninfected controls (black), “moderate” COVID-19 (defined as WHO grade 3 in corresponding publication (blue) and “critical” COVID-19 (WHO grade 6-7 in corresponding publication) (red). Repartition of data points for each cell type if not stated otherwise: control n=5/5 (individuals/time points), moderate n=8/14, critical n=11/13. Thick lines indicate smoothed population trends based on a LOESS estimate, thin lines connect subjects. shaded areas indicate 95% CI for the LOESS estimate.

(A) Ionocytes control n=3/3, Secretory cells critical n=10/12, Secretory cells – differentiating critical n=10/12, Squamous cells critical n=9/11.

(B) B cells control n=3/3, moderate n=7/13, critical n=10/11, cytotoxic T cells (CTL) control n=4/4, MD dendritic cells control n=1/1, NKT cells control n=2/2, moderate n=7/13, Plasmacytoid dendritic cells control n=1/1, moderate n=7/13, critical n=8/10, Regulatory T cells control n=4/4, critical n=10/12, critical n=10/12.


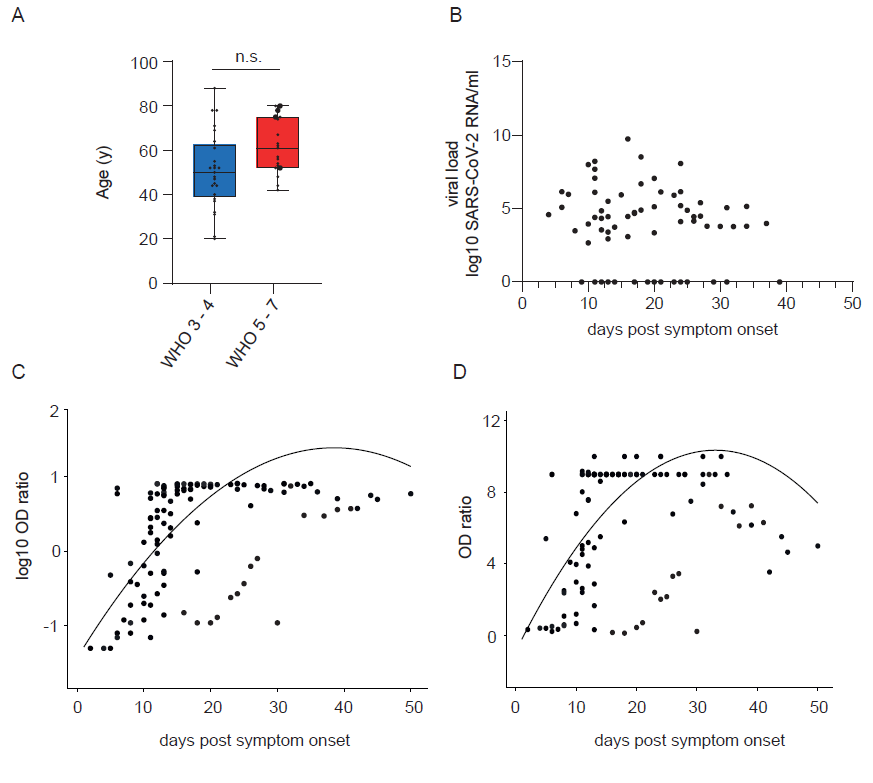


***Suppl. Fig. 5* Characterisation of COVID-19 Patients**

(A) COVID-19 disease severity and age of infected individuals. WHO 3-4 n=25, WHO 5-7 n=19. Large points indicate deceased patients. Unpaired t-test (p=0.15).

(B) SARS-CoV-2 RNA concentrations in COVID-19 cohort after symptom onset. n=32, 68 time points.

(C) SARS-CoV-2 IgG antibody levels in COVID-19 cohort after symptom onset. n=40, 113 samples at different time points. Log-transformed OD ratios were modelled using a linear mixed effects model, with days post symptom onset (with both a linear and a quadratic term) as fixed effect and patient ID as random intercept. The resulting regression line is indicated (R^2^ = 0.86).

(D) SARS-CoV-2 IgA antibody levels in COVID-19 cohort after symptom onset. n=40, 113 samples at different time points. OD ratios were modelled using a linear mixed effects model, with days post symptom onset (with both a linear and a quadratic term) as fixed effect and patient ID as random intercept. The resulting regression line is indicated (R^2^ = 0.78).

**
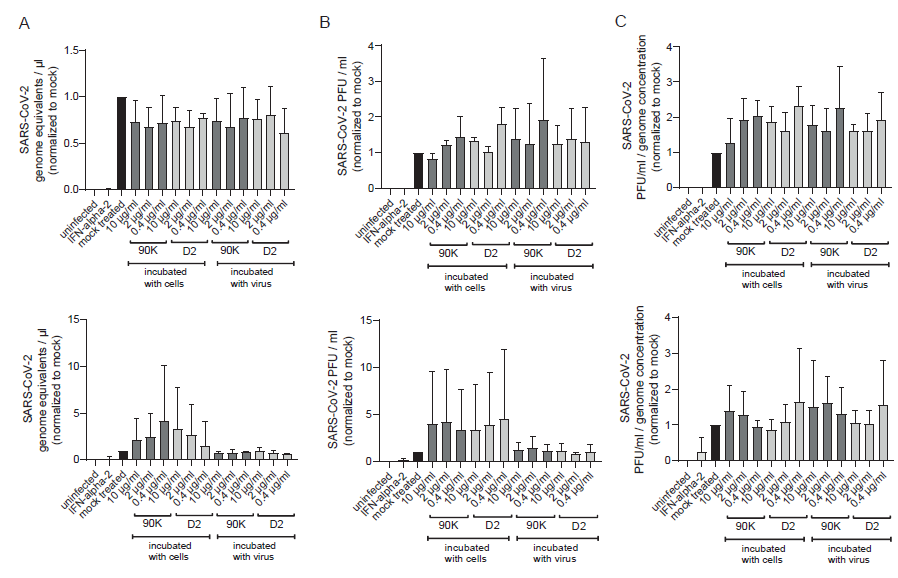
**

***Suppl. Fig. 6* SARS-CoV-2 Particle Release and Infectivity Remains Intact Upon Addition of Exogenous 90K or 90K-D2**

Calu-3 (upper graphs) and Caco-2 (lower graphs) cells or virus stocks were treated with indicated concentrations of purified, full-length 90K and 90K(D2) or left untreated at 37°C for two hours prior to infection. 24 hours post-infection, supernatant was harvested for quantification of SARS-CoV-2 genome equivalents/ul (A) and plaque assays PFU/ml (B) in Vero E6 cells. Data are shown normalized to mock. Particle infectivity was calculated by normalizing SARS-CoV-2 PFU to the genome equivalents/µl, also depicted normalized to mock (C). Results arise from three independent experiments.


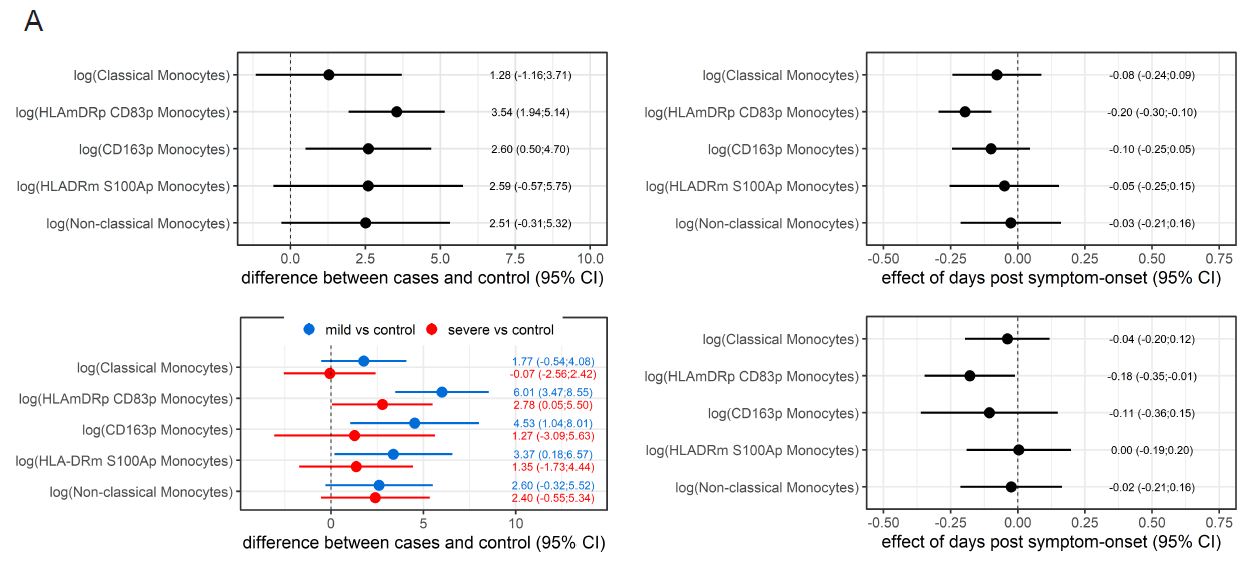


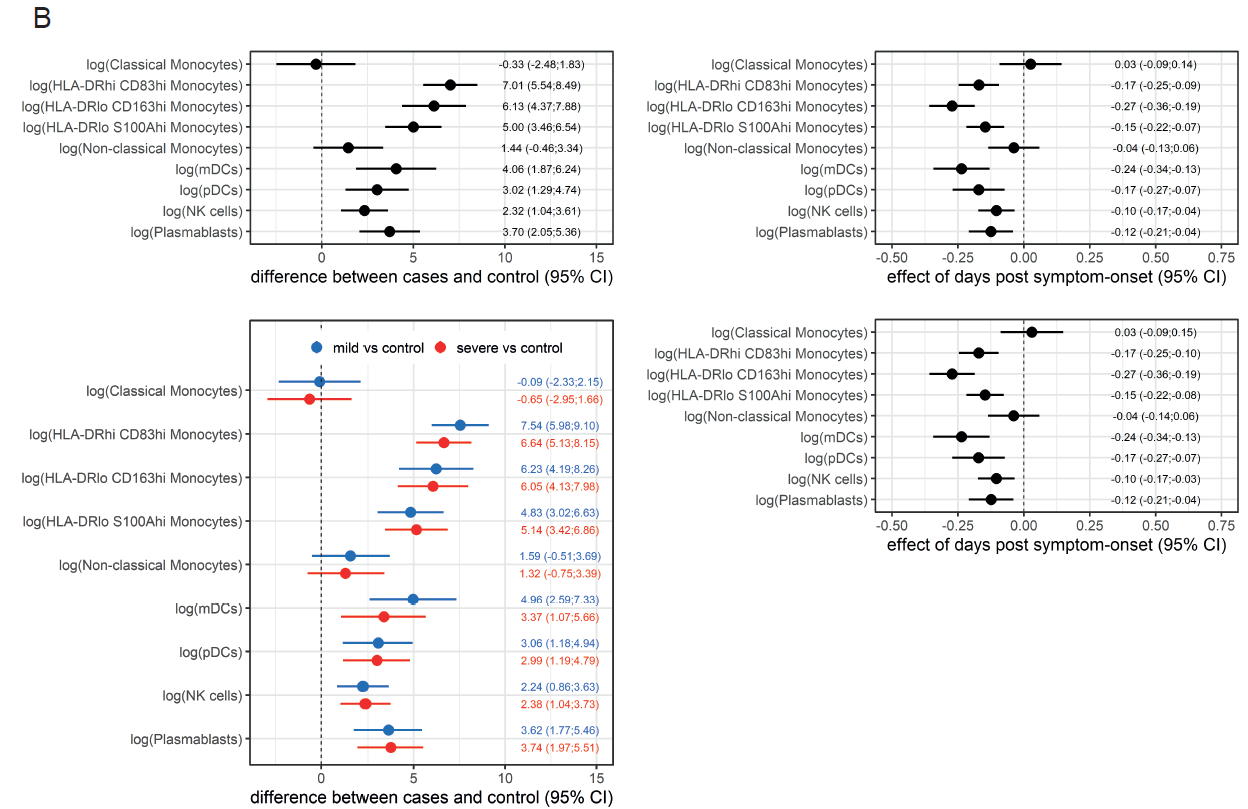


***Suppl. Fig. 7* Statistical Analyses of scRNA-seq PBMC Datasets**

Forest plots of analyzed scRNA-seq PBMC datasets from cohort A (A) and cohort B (B). Whiskers indicate 95% CI, derived from mixed linear regression models for two (cases vs. controls, impact of days post symptom onset) and three groups (mild cases vs. controls, severe cases vs. controls, impact of days post symptom onset).


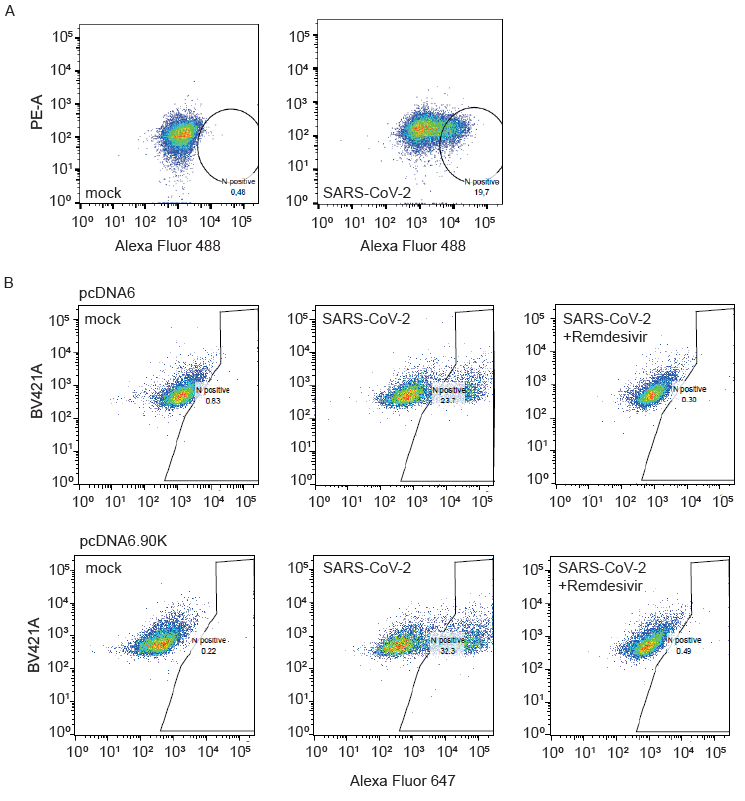


***Suppl. Fig. 8* SARS-CoV-2 Infection Rates in Used Cell Lines**

(A) SARS-CoV-2 intracellular nucleocapsid stain in Calu-3 cells. Cells were infected at MOI 0.01 and harvested 48 hours post infection for flow cytometry. Cells were stained for Nucleocapsid followed by detection with a secondary antibody conjugated to Alexa Fluor 488.

(B) SARS-CoV-2 intracellular nucleocapsid stain in Caco-2 cells transduced with empty vector (pcDNA6) or 90K (pcDNA6.90K). Cells were infected at MOI 0.01 and harvested 24 hours post infection for flow cytometry. Cells were first stained for intracellular nucleocapsid and detected with a secondary antibody conjugated to Alexa Fluor 477. Remdesivir treatment (20uM) was used for inhibition of infection as negative control.


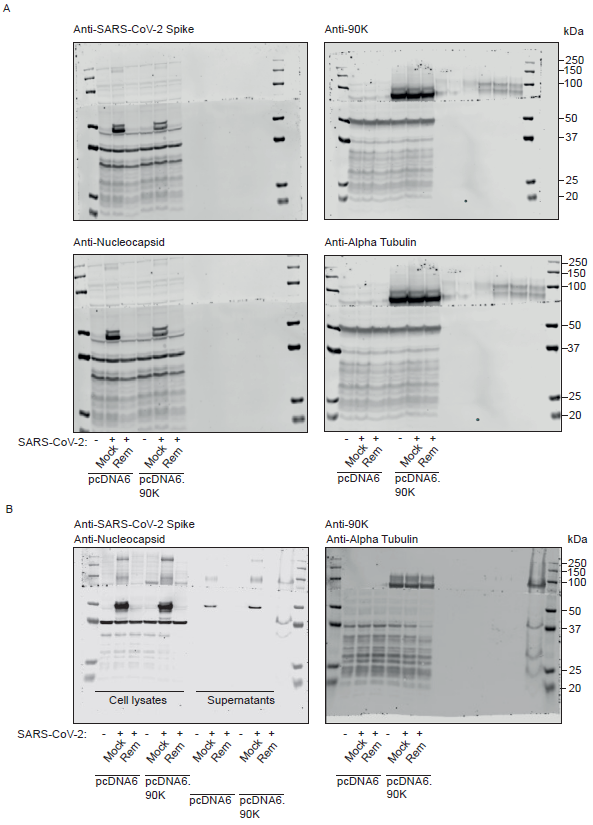


***Suppl. Fig. 9* Raw Data From Immunoblots Related to Figure 6**

(A) Raw immunoblot membrane visualization data related to Figure 6A

(B) Raw immunoblot membrane visualization data related to Figure 6B

| **Patient N°** | **Sample** | **90K Serum ELISA** | **90K Serum Level from ELISA in µg/ml** | **90K PBMC ELISA Levels in µg/ml** | ***LGALS3BP* qRT PCR** | **WHO Grade** | **Sex** | **Age (y)** | **Days post Symptom Onset** |
| --- | --- | --- | --- | --- | --- | --- | --- | --- | --- |
| **1** | **A** | **X** | **10.80** |  |  | **3** | **M** | **21** | **16** |
| **1** | **B** | **X** | **8.60** |  |  | **3** | **M** | **21** | **18** |
| **1** | **C** | **X** | **11.96** |  |  | **3** | **M** | **21** | **20** |
| **1** | **D** | **X** | **11.65** |  |  | **3** | **M** | **21** | **21** |
| **1** | **E** | **X** | **11.15** |  |  | **3** | **M** | **21** | **23** |
| **1** | **F** | **X** | **7.18** |  |  | **3** | **M** | **21** | **24** |
| **1** | **G** | **X** | **9.13** |  |  | **3** | **M** | **21** | **25** |
| **1** | **H** | **X** | **9.22** |  |  | **3** | **M** | **21** | **26** |
| **1** | **I** | **X** | **7.60** |  |  | **3** | **M** | **21** | **27** |
| **1** | **J** | **X** | **17.82** |  |  | **3** | **M** | **21** | **30** |
| **1** | **K** | **X** | **25.63** |  |  | **3** | **M** | **21** | **32** |
| **1** | **L** | **X** | **6.68** |  |  | **3** | **M** | **21** | **34** |
| **1** | **M** | **X** | **5.61** |  |  | **3** | **M** | **21** | **37** |
| **1** | **N** | **X** | **6.21** |  |  | **3** | **M** | **21** | **39** |
| **1** | **O** | **X** | **6.09** |  |  | **3** | **M** | **21** | **41** |
| **2** | **A** | **X** | **13.51** |  |  | **3** | **F** | **31** | **8** |
| **2** | **B** | **X** | **11.23** |  |  | **3** | **F** | **31** | **10** |
| **2** | **C** | **X** | **10.92** |  |  | **3** | **F** | **31** | **11** |
| **2** | **D** | **X** | **10.26** |  |  | **3** | **F** | **31** | **12** |
| **2** | **E** | **X** | **8.56** |  |  | **3** | **F** | **31** | **13** |
| **3** | **A** | **X** | **22.38** |  |  | **3** | **M** | **45** | **11** |
| **3** | **B** | **X** | **23.09** |  |  | **3** | **M** | **45** | **12** |
| **3** | **C** | **X** | **20.90** |  |  | **3** | **M** | **45** | **13** |
| **3** | **D** | **X** | **17.31** |  |  | **3** | **M** | **45** | **16** |
| **3** | **E** | **X** | **5.65** |  |  | **3** | **M** | **45** | **18** |
| **3** | **F** | **X** | **19.03** |  |  | **3** | **M** | **45** | **20** |
| **3** | **G** | **X** | **14.40** |  |  | **3** | **M** | **45** | **23** |
| **3** | **H** | **X** | **12.31** |  |  | **3** | **M** | **45** | **25** |
| **4** | **A** | **X** | **28.85** |  |  | **4** | **M** | **71** | **13** |
| **4** | **B** | **X** | **23.85** |  |  | **4** | **M** | **71** | **14** |
| **4** | **C** | **X** | **33.28** |  |  | **4** | **M** | **71** | **17** |
| **4** | **D** | **X** | **18.08** |  |  | **4** | **M** | **71** | **19** |
| **5** | **A** | **X** | **15.23** |  |  | **4** | **M** | **32** | **8** |
| **5** | **B** | **X** | **14.70** |  |  | **4** | **M** | **32** | **11** |
| **5** | **C** | **X** | **16.71** |  |  | **4** | **M** | **32** | **12** |
| **5** | **D** | **X** | **9.00** |  |  | **4** | **M** | **32** | **15** |
| **5** | **E** | **X** | **11.16** |  |  | **4** | **M** | **32** | **17** |
| **6** | **A** | **X** | **6.13** |  |  | **7** | **M** | **63** | **24** |
| **6** | **B** | **X** | **16.41** |  |  | **7** | **M** | **63** | **26** |
| **6** | **C** | **X** | **26.26** |  |  | **7** | **M** | **63** | **28** |
| **6** | **D** | **X** | **17.44** |  |  | **7** | **M** | **63** | **31** |
| **6** | **E** | **X** | **7.55** |  |  | **7** | **M** | **63** | **33** |
| **6** | **F** | **X** | **14.09** |  |  | **7** | **M** | **63** | **35** |
| **7** | **A** | **X** | **19.33** |  |  | **6** | **F** | **80** | **6** |
| **7** | **B** | **X** | **23.75** |  |  | **6** | **F** | **80** | **8** |
| **7** | **C** | **X** | **27.77** |  |  | **6** | **F** | **80** | **11** |
| **7** | **D** | **X** | **54.76** |  |  | **6** | **F** | **80** | **13** |
| **7** | **E** | **X** | **14.47** |  |  | **6** | **F** | **80** | **15** |
| **8** | **A** | **X** | **19.46** |  |  | **3** | **F** | **50** | **10** |
| **8** | **B** | **X** | **22.89** |  |  | **3** | **F** | **50** | **11** |
| **9** | **A** | **X** | **16.51** |  |  | **5** | **M** | **61** | **12** |
| **9** | **B** | **X** | **38.00** |  |  | **5** | **M** | **61** | **17** |
| **9** | **C** | **X** | **19.31** |  |  | **5** | **M** | **61** | **19** |
| **10** | **A** | **X** | **20.01** |  |  | **6** | **M** | **75** | **12** |
| **10** | **B** | **X** | **42.36** |  |  | **6** | **M** | **75** | **13** |
| **10** | **C** | **X** | **26.38** |  |  | **6** | **M** | **75** | **16** |
| **10** | **D** | **X** | **17.77** |  |  | **6** | **M** | **75** | **18** |
| **10** | **E** | **X** | **41.86** |  |  | **6** | **M** | **75** | **20** |
| **11** | **A** | **X** | **39.34** |  |  | **7** | **M** | **52** | **8** |
| **12** | **A** | **X** | **22.66** |  |  | **6** | **M** | **74** | **4** |
| **12** | **B** | **X** | **25.86** |  |  | **6** | **M** | **74** | **6** |
| **12** | **C** | **X** | **54.15** |  |  | **6** | **M** | **74** | **8** |
| **13** | **A** | **X** | **19.90** |  |  | **3** | **F** | **61** | **21** |
| **14** | **A** | **X** | **16.38** |  |  | **3** | **M** | **52** | **9** |
| **14** | **B** | **X** | **15.77** |  |  | **3** | **M** | **52** | **11** |
| **14** | **C** | **X** | **15.35** |  |  | **3** | **M** | **52** | **13** |
| **15** | **A** | **X** | **18.59** |  |  | **6** | **F** | **53** | **11** |
| **15** | **B** | **X** | **23.03** |  |  | **6** | **F** | **53** | **13** |
| **15** | **C** | **X** | **32.68** |  |  | **6** | **F** | **53** | **15** |
| **16** | **A** | **X** | **21.88** |  |  | **6** | **M** | **52** | **11** |
| **16** | **B** | **X** | **17.45** |  |  | **6** | **M** | **52** | **13** |
| **16** | **C** | **X** | **9.83** |  |  | **6** | **M** | **52** | **15** |
| **17** | **A** | **X** | **20.58** |  |  | **5** | **M** | **62** | **14** |
| **17** | **B** | **X** | **22.04** |  |  | **5** | **M** | **62** | **16** |
| **17** | **C** | **X** | **17.20** |  |  | **5** | **M** | **62** | **18** |
| **18** | **A** | **X** | **49.96** |  |  | **3** | **F** | **44** | **asymptomatic** |
| **18** | **B** | **X** | **17.63** |  |  | **3** | **F** | **44** | **asymptomatic** |
| **18** | **C** | **X** | **21.14** |  |  | **3** | **F** | **44** | **asymptomatic** |
| **19** | **A** | **X** | **9.03** |  |  | **4** | **M** | **64** | **12** |
| **19** | **B** | **X** | **8.68** |  |  | **4** | **M** | **64** | **14** |
| **20** | **A** | **X** | **27.11** |  |  | **5** | **M** | **56** | **10** |
| **20** | **B** | **X** | **50.44** |  |  | **5** | **M** | **56** | **12** |
| **21** | **A** | **X** | **12.50** |  |  | **3** | **F** | **40** | **13** |
| **22** | **A** | **X** | **27.91** |  |  | **4** | **M** | **53** | **12** |
| **22** | **B** | **X** | **25.39** |  |  | **4** | **M** | **53** | **14** |
| **23** | **A** | **X** | **11.31** |  |  | **3** | **M** | **47** | **5** |
| **24** | **A** | **X** | **21.59** |  |  | **3** | **F** | **37** | **12** |
| **25** | **A** | **X** | **8.34** |  |  | **3** | **M** | **78** | **13** |
| **26** | **A** | **X** | **13.34** | **0.18** | **X** | **4** | **M** | **78** | **20** |
| **27** | **A** | **X** | **31.99** | **0.74** | **X** | **3** | **F** | **69** | **6** |
| **28** | **A** | **X** | **48.14** |  |  | **4** | **M** | **55** | **6** |
| **29** | **A** | **X** | **27.93** |  | **X** | **3** | **F** | **20** | **15** |
| **30** | **A** | **X** | **30.66** |  |  | **3** | **F** | **38** | **2** |
| **31** | **A** | **X** | **27.23** | **0.12** | **X** | **7** | **M** | **67** | **29** |
| **31** | **B** | **X** | **21.44** |  |  | **7** | **M** | **67** | **36** |
| **31** | **C** | **X** | **18.71** |  |  | **7** | **M** | **67** | **44** |
| **31** | **D** | **X** | **30.82** |  |  | **7** | **M** | **67** | **50** |
| **32** | **A** | **X** | **15.82** | **0.19** | **X** | **7** | **M** | **78** | **24** |
| **32** | **B** | **X** | **8.58** | **0.12** |  | **7** | **M** | **78** | **31** |
| **32** | **C** | **X** | **15.57** |  | **X** | **7** | **M** | **78** | **39** |
| **32** | **D** | **X** | **11.11** |  |  | **7** | **M** | **78** | **45** |
| **32** | **E** | **X** | **12.66** | **0.63** | **X** | **7** | **M** | **78** | **42** |
| **33** | **A** | **X** | **59.64** |  |  | **5** | **M** | **48** | **5** |
| **34** | **A** | **X** | **73.81** | **0.31** | **X** | **4** | **W** | **88** | **7** |
| **35** | **A** | **X** | **42.68** | **0.27** | **X** | **3** | **M** | **52** | **13** |
| **36** | **A** | **X** | **28.74** | **0.84** | **X** | **6** | **M** | **44** | **17** |
| **36** | **B** | **X** | **18.19** | **0.15** |  | **6** | **M** | **44** | **24** |
| **36** | **C** | **X** | **20.95** | **0.4** |  | **6** | **M** | **44** | **31** |
| **37** | **A** | **X** | **23.99** |  |  | **7** | **M** | **57** | **10** |
| **37** | **B** | **X** | **11.67** | **0.25** | **X** | **7** | **M** | **57** | **17** |
| **37** | **C** | **X** | **10.05** |  | **X** | **7** | **M** | **57** | **24** |
| **38** | **A** | **X** | **20.39** |  | **X** | **4** | **M** | **52** | **11** |
| **38** | **B** | **X** | **24.68** | **1.35** | **X** | **4** | **M** | **52** | **18** |
| **39** | **A** | **X** | **11.46** |  |  | **4** | **M** | **44** | **28** |
| **40** | **A** | **X** | **13.36** | **0.23** | **X** | **3** | **M** | **48** | **10** |
| **41** | **A** | **X** | **50.90** |  | **X** | **7** | **M** | **54** | **13** |
| **41** | **B** | **X** | **38.52** |  |  | **7** | **M** | **54** | **20** |
| **42** | **A** | **X** | **37.60** | **0.15** | **X** | **7** | **M** | **75** | **27** |
| **42** | **B** | **X** | **17.83** | **0.34** | **X** | **7** | **M** | **75** | **34** |
| **43** | **A** |  |  |  | **X** | **5** | **W** | **42** | **16** |
| **44** | **A** |  |  | **0.75** |  | **5** | **W** | **80** | **10** |

***Suppl. Table 1* Overview of Sampled Data in COVID-19 Cohort**

Each patient sample is attributed to sample type, disease severity, sex (M = male, F = female), age in years and days post symptom onset on sampling day.

| Matched healthy controls  n=42 serum samples  Age (y) | Sex | Age section (y) | COVID-19 Cohort  n=42 serum samples  Age (y) |
| --- | --- | --- | --- |
| **31** | **M** | **25-34** | **31** |
| **38** | **F** | **35-44** | **38** |
| **69** | **M** | **65 +** | **75** |
| **67** | **M** | **65 +** | **67** |
| **48** | **M** | **45-54** | **48** |
| **50** | **F** | **45-54** | **50** |
| **44** | **M** | **35-44** | **44** |
| **51** | **M** | **45-54** | **52** |
| **69** | **M** | **65 +** | **78** |
| **70** | **M** | **65 +** | **71** |
| **52** | **M** | **45-54** | **52** |
| **37** | **F** | **35-44** | **37** |
| **22** | **M** | **< 25** | **21** |
| **68** | **F** | **65 +** | **68** |
| **57** | **M** | **55-64** | **56** |
| **44** | **F** | **35-44** | **44** |
| **54** | **M** | **45-54** | **52** |
| **64** | **M** | **55-64** | **64** |
| **53** | **M** | **45-54** | **52** |
| **61** | **M** | **55-64** | **61** |
| **67** | **F** | **65 +** | **69** |
| **69** | **M** | **65 +** | **78** |
| **44** | **M** | **35-44** | **44** |
| **31** | **F** | **25-34** | **31** |
| **53** | **F** | **45-54** | **53** |
| **24** | **F** | **< 25** | **20** |
| **49** | **M** | **45-54** | **54** |
| **48** | **M** | **45-54** | **48** |
| **68** | **F** | **65 +** | **88** |
| **53** | **M** | **45-54** | **52** |
| **58** | **M** | **55-64** | **55** |
| **62** | **M** | **55-64** | **62** |
| **45** | **M** | **45-54** | **45** |
| **47** | **M** | **45-54** | **47** |
| **69** | **M** | **65 +** | **78** |
| **68** | **M** | **65 +** | **74** |
| **63** | **M** | **55-64** | **63** |
| **70** | **M** | **65 +** | **75** |
| **58** | **M** | **55-64** | **57** |
| **54** | **M** | **45-54** | **53** |
| **64** | **F** | **55-64** | **61** |
| **42** | **F** | **35-44** | **40** |

***Suppl. Table 2* Age and Sex Match Between Healthy Controls and COVID-19 Patients**

Each patient is attributed to one healthy control, matched in age categories and sex (M = male, F = female).

**Supplemental References**

[50. Dobin A, Davis CA, Schlesinger F et al. STAR: ultrafast universal RNA-seq aligner. Bioinformatics [Internet]. 2013;29:15–21. Available from:](http://paperpile.com/b/oA8C2P/tymG) <http://dx.doi.org/10.1093/bioinformatics/bts635>

[51. Hao Y, Hao S, Andersen-Nissen E, et al. Integrated analysis of multimodal single-cell data. Cell [Internet]. 2021;184:3573–87.e29. Available from:](http://paperpile.com/b/oA8C2P/iHbv) <http://dx.doi.org/10.1016/j.cell.2021.04.048>

[52. Okba NMA, Müller MA, Li W et al. Severe Acute Respiratory Syndrome Coronavirus 2-Specific Antibody Responses in Coronavirus Disease Patients. Emerg Infect Dis [Internet]. 2020;26:1478–88. Available from:](http://paperpile.com/b/oA8C2P/kNDMb) <http://dx.doi.org/10.3201/eid2607.200841>

[53. Mühlemann B, Thibeault C, Hillus D et al. Impact of dexamethasone on SARS-CoV-2 concentration kinetics and antibody response in hospitalized COVID-19 patients: results from a prospective observational study. Clin Microbiol Infect [Internet]. 2021;27:1520.e7–1520.e10. Available from:](http://paperpile.com/b/oA8C2P/7xG8) <http://dx.doi.org/10.1016/j.cmi.2021.06.008>

[54. Corman VM, Landt O, Kaiser M et al. Detection of 2019 novel coronavirus (2019-nCoV) by real-time RT-PCR. Euro Surveill [Internet]. 2020;25. Available from:](http://paperpile.com/b/oA8C2P/AbpWG) <http://dx.doi.org/10.2807/1560-7917.ES.2020.25.3.2000045>

[55. Jones TC, Biele G, Mühlemann B et al. Estimating infectiousness throughout SARS-CoV-2 infection course. Science [Internet]. 2021;373. Available from:](http://paperpile.com/b/oA8C2P/y3clc) <http://dx.doi.org/10.1126/science.abi5273>

[56. Niemeyer D, Stenzel S, Veith T et al. SARS-CoV-2 variant Alpha has a spike-dependent replication advantage over the ancestral B.1 strain in human cells with low ACE2 expression. PLoS Biol [Internet]. 2022;20:e3001871. Available from:](http://paperpile.com/b/oA8C2P/Wy2e) <http://dx.doi.org/10.1371/journal.pbio.3001871>
